# The mycobiome, virome and archaeome in gastrointestinal cancers: molecular pathogenesis and therapeutic intervention

**DOI:** 10.1186/s12943-026-02698-3

**Published:** 2026-05-28

**Authors:** Cillian H. Cheng, Chi Chun Wong

**Affiliations:** Institute of Digestive Disease and Department of Medicine and Therapeutics, State Key Laboratory of Digestive Disease, Li Ka Shing Institute of Health Sciences, The Chinese University of Hong Kong, Hong Kong SAR, China

**Keywords:** Gastrointestinal (GI) cancer, Mycobiome, Virome, Archaeome, Cancer development, Clinical translation

## Abstract

Gastrointestinal (GI) cancers remain a significant global health challenge. For decades, research has concentrated on the bacterial microbiome’s role in tumour development, largely neglecting the important roles of the non-bacterial kingdoms, including mycobiome (fungi), the virome (viruses), and archaeome (archaea). These elements represent an underexplored and crucial “dark matter” of the microbiome. This review aims to systematically summarize current evidence on the compositional alterations of viruses, fungi, and archaea across the major types of GI cancer, including colorectal, hepatocellular, gastric, pancreatic and esophageal/oral cancers. We critically examine how viruses, fungi, and archaea directly affect host cellular processes and indirectly influence cancer risk through complex cross-kingdom interactions with the bacterial microbiota and the host immune system. Additionally, we explore the significant translational potential of this knowledge, emphasizing opportunities to use these non-bacterial communities in developing new diagnostic biomarkers and therapeutic strategies. Finally, we highlight the importance of future multi-kingdom integrative analyses to fully understand the microbial ecosystem involved in GI oncogenesis and to translate these insights into clinical practice.

## Introduction

Gastrointestinal (GI) cancers are leading causes of morbidity and mortality worldwide [1] and human GI tract harbors a complex ecosystem of microorganisms, long recognized as a critical determinant of health and disease. For decades, research has predominantly focused on the bacterial component of this microbiome, unveiling its profound influence on host physiology, immunity, and carcinogenesis [2–10]. However, this represents a limited view of a far more intricate biological landscape. A diverse consortium of non-bacterial microorganisms, including fungi, viruses, and archaea, coexists within the GI tract, constituting what is now acknowledged as the “non-bacterial microbiome”. Despite their lower relative abundance, these kingdoms exert a disproportionate impact on ecological dynamics and host pathophysiology. The gut mycobiome, although constituting less than 0.1% of microbial cells, represents a significant source of bioactive molecules owing to the substantial size of fungal cells. The virome, predominantly composed of bacteriophages, exerts a significant influence on bacterial communities and, via eukaryotic viruses, exhibits direct oncogenic potential. Meanwhile, the archaeome, especially methanogens, assumes a crucial role in sustaining metabolic homeostasis. Although the gut protistome, including protozoa and other eukaryotic parasites, represents another important component of the non-bacterial eukaryotic microbiome, its role in GI carcinogenesis is far less characterized, with limited mechanistic and translational evidence. Therefore, the present review focuses on the mycobiome, virome, and archaeome, which have garnered substantial functional and clinical insights in recent years. Collectively, these components establish an integrated, trans-kingdom network increasingly associated with the initiation and progression of GI cancers.

Recent technological and conceptual advances have propelled the non-bacterial microbiome from a neglected frontier to a central area of oncological research. Mounting evidence reveals that specific fungi, such as *Candida* and *Malassezia*, can promote tumorigenesis by inducing chronic inflammation, modulating immune responses, and producing genotoxic metabolites. Oncogenic viruses, including Epstein-Barr virus (EBV) and hepatitis viruses, are established etiological agents in a subset of gastric and liver cancers, respectively. Even the enigmatic archaea are now linked to colorectal carcinogenesis through stage-specific ecological restructuring. This burgeoning body of work underscores that a comprehensive understanding of GI cancer is incomplete without considering the concerted and individual roles of these non-bacterial players.

Importantly, these trans-kingdom dynamics are gaining increased significance within the context of a shifting demographic landscape. There is an alarming, global rise in early-onset GI cancers, particularly colorectal and pancreatic cancers affecting individuals under the age of 50, a trend not fully accounted for by genetics or diet [11]. Emerging evidence links early-onset colorectal cancer to gut microbial dysbiosis, including pro-inflammatory and genotoxic taxa, interacting with host genetics, immunity, and early-life exposures to drive carcinogenesis [12]. Consequently, understanding the potential oncogenic roles of fungi, viruses, and archaea is not only fundamental to tumour biology but also critical for addressing the specific vulnerabilities of the rising early-onset patient population.

In this review, we provide an overview of current knowledge regarding the non-bacterial microbiome in GI oncology. Initially, it provides an overview of the mycobiome, virome, and archaeome within the human gut. Subsequently, it elucidates their associations and mechanistic roles across major GI malignancies, including colorectal, hepatocellular, gastric, pancreatic, and esophageal cancers. Special emphasis is placed on clarifying the molecular mechanisms, such as immunomodulation, metabolite production, genotoxicity, and cross-kingdom interactions, that underpin their pro-tumorigenic effects. Finally, the discussion encompasses the significant translational potential of this field, exploring how these microbes function as novel diagnostic and prognostic biomarkers, and examining how therapeutic strategies targeting or exploiting them, including antifungals, phage therapy, and probiotics, are heralding a new chapter in precision cancer therapy.

## The non-bacterial microbiome: an overview

### Mycobiome

Despite constituting less than 0.1% of the gut’s microbial inhabitants, the gut mycobiome represents a pivotal and functionally distinct component of the human microbiome [13]. Its uniqueness is underscored by the fact that fungal cells are approximately 100 times larger than their bacterial counterparts, enabling them to be a significant source of bioactive molecules that influence host physiology [14, 15]. The human gut harbors a remarkable diversity of fungi, with at least 267 distinct taxa identified, including genera such as *Candida*, *Saccharomyces*, and *Aspergillus* [16, 17]. This community is composed of a core set of approximately 200 common species and a highly variable component of over 800 species, the composition of which is profoundly shaped by geography, diet, and health status [18–20]. The phyla Ascomycota and Basidiomycota typically dominate this ecosystem in healthy adults [21]. Notably, the gut mycobiome exhibits greater inter-individual variability and temporal instability than the bacterial microbiome, highlighting its dynamic nature [18].

The development of the gut mycobiome is a staged process, intricately linked to host maturation and dietary shifts. In healthy infants, initial colonization by Saccharomycetales and *Malassezia* is followed by a rapid decline within the first five months [22, 23]. Subsequently, the introduction of solid foods drives a shift toward a community dominated by *Saccharomyces cerevisiae* and *Cystoflobasidium* spp., Ascomycota spp., and *Monographella* spp., thereby emphasizing diet as a fundamental determinant of mycobiome composition [22]. This trajectory persists into adulthood, where microbial diversity expands and is predominantly characterized by the dominance of Ascomycota, Basidiomycota, and Zygomycota [17, 24]. Furthermore, host-intrinsic factors such as sex and age are key determinants [20]. For instance, *Aspergillus* is more frequent in males, while *Candida* is often enriched in females, and fungal diversity generally decreases with advancing age [25, 26]. Moving beyond correlative associations, recent studies are beginning to elucidate potential mechanistic links between fungal activity and oncogenic processes, marking a critical evolution in the field [27–30].

### Virome

The human gut virome represents a substantial component of the GI ecosystem, with densities reaching up to 10^9^ virus-like particles (VLPs) per gram of fecal content [31, 32]. It is predominantly comprised of bacteriophages (phages), viruses that specifically target bacteria. Phages exhibit essential viral features, including their diminutive size, acellular composition, and presence of a single type of nucleic acid [33]. Their life cycle is primarily characterized by two modes: the lytic cycle, which causes the immediate lysis and demise of the bacterial host, and the lysogenic cycle, whereby the phage genome becomes integrated into the host chromosome as a prophage, subsequently replicating passively alongside the bacterium [34]. This dynamic interplay profoundly influences bacterial community structure and function.

The composition of the virome is not static but undergoes significant evolution throughout the lifespan. During infancy, phage populations are highly dynamic and reflect the rapid development of the bacterial microbiome [35]. Recent data from 2025 indicate that, in one-year-old infants, approximately 70% of viral Operational Taxonomic Units (vOTUs) are classified into five main categories: *Arfiviricetes*, *Caudoviricetes*, *Faserviricetes*, *Malgrandaviricetes*, and *Tectiliviricetes* [36]. This initial community ultimately consolidates into a more stable adult core virome, characterized by the predominance of *Crassvirales* (class *Caudoviricetes*) and *Microviridae* (class *Malgrandaviricetes*) [31, 37, 38]. Beyond host development, environmental factors such as diet assume a pivotal role. Western diets, characterized by high fat content and low fiber intake, have been demonstrated to adversely affect phage diversity and functionality. These alterations mirror the dysbiotic bacterial shifts observed in metabolic and inflammatory conditions [39]. Critically, the influence of gut virome extends to carcinogenesis. In addition to the modulatory role of phages, which can promote bacterial evolution and dysbiosis, the direct oncogenic potential has been established for several eukaryotic viruses. Pathogens such as Epstein-Barr virus (EBV) [40, 41], human papillomavirus (HPV) [42, 43], hepatitis viruses [44, 45], and severe acute respiratory syndrome coronavirus 2 (SARS-CoV-2) [46, 47] have been identified in patients with GI cancers. Therefore, the virome constitutes an essential component of the gut microbiome, capable of influencing host physiology and disease both indirectly through bacterial interactions and directly via oncogenic mechanisms.

### Archaeome

Archaea, comprising the third domain of life, represent a distinct group of prokaryotes that have been reclassified from merely inhabiting extreme environments to becoming integral constituents of the human microbiome [48, 49]. Although traditionally recognized for thriving in extreme niches because of their distinctive cellular composition, characterized by the absence of peptidoglycan, esters, and fatty acids, advances in sequencing technologies have now definitively confirmed their presence across multiple human body sites, including the GI tract [50–52]. Within the gut, archaea constitute up to 4% of the microbial community and are predominantly represented by methanogens, with *Methanobrevibacter* and *Methanosphaera* identified as the most prevalent genera [53, 54]. Despite their relatively low abundance, the gut archaeome plays a disproportionately crucial role in maintaining intestinal homeostasis primarily through methanogenesis [55]. This process is not merely a metabolic curiosity but a fundamental ecosystem service. By consuming bacterial fermentation end-products, such as hydrogen and acetates, methanogens facilitate more efficient microbial metabolism and help prevent the detrimental accumulation of these compounds [54]. This functional integration extends beyond methanogenesis to include roles in carbohydrate and trimethylamine metabolism, as well as immunomodulation, positioning archaea as key modulators of the gut environment [56, 57].

The establishment of this advantageous community initiates early in life. Evidence of methanogenic archaea in the colostrum and breast milk of healthy women indicates a natural mechanism for vertical transmission, inoculating the infant gut with these vital microbes [58]. Consequently, archaea are recognized not as obsolete remnants or incidental occupants but as essential symbiotic partners that actively contribute to the stability and functionality of the human GI ecosystem. Nevertheless, despite their significant role, archaea have been largely neglected in studies dissecting host-associated microbial communities in GI cancer. This oversight represents a critical gap in our understanding of oncobiosis and tumorigenesis.

## Role in specific gastrointestinal cancers

**Fig. 1:**
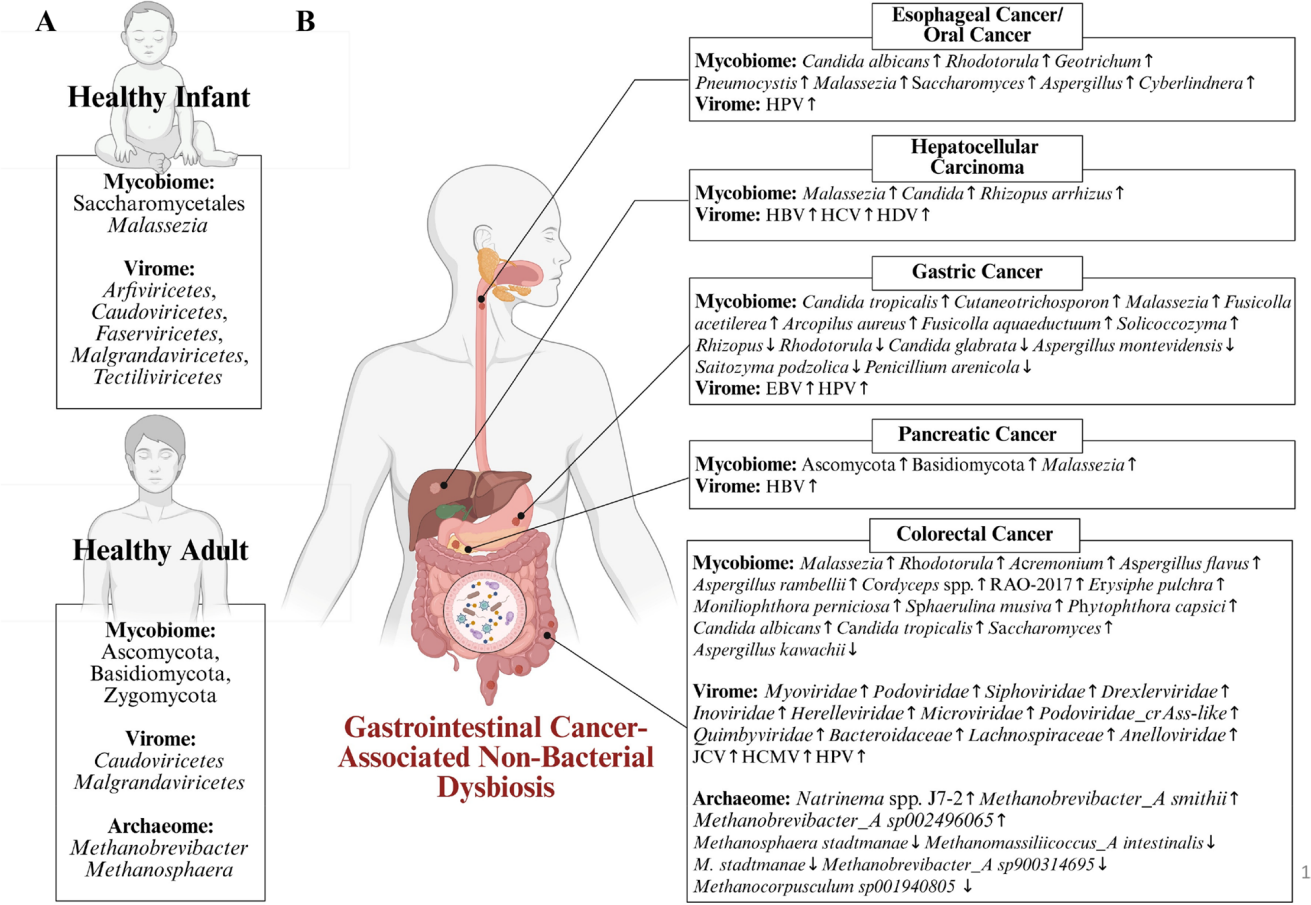
Developmental trajectory and dysregulated state of the gastrointestinal microbiome. **A** Maturation trajectory of the non-bacterial gut mycobiome, virome, and archaeome. **B** A schematic overview of cross-kingdom microbial dysbiosis associated with major gastrointestinal cancers. EBV, Epstein–Barr virus, JCV, John Cunningham virus; HBV, hepatitis B virus; HCMV, human cytomegalovirus; HCV, hepatitis C virus; HDV, hepatitis D virus; HPV, human papillomavirus

The fungal, viral, and archaeal constituents of the microbiome are now acknowledged as pivotal functional contributors to GI carcinogenesis. They impact cancer initiation and progression through direct interactions with the host, immunomodulatory effects, and remodeling of the tumour microenvironment (TME), thereby establishing the multikingdom microbiota as a significant oncogenic regulator (Fig. 1 and Table 1).

**Table 1:** Summary of non-bacterial microbial influences in gastrointestinal cancers

Cancer type	Microbial domain	Representative species	Overall effect on carcinogenesis	Mechanism of action	Level of evidence
Colorectal cancer	Fungi	Enriched in CRC: *Aspergillus flavus*, *Malassezia globosa*, *Rhodotorula* spp., *Acremonium* spp., *Moniliophthora perniciosa* Depleted in CRC: *Saccharomyces cerevisiae*, *Lipomyces starkeyi*	Promotive (for enriched species) Protective (for depleted species)	1. Potential toxin production (e.g., Aflatoxin by *A. flavus*) 2. Immune modulation (suggested for depleted *S. cerevisiae*)	Humans
	Fungi	*Trichosporon* spp., *Malassezia* spp.	Promotive	Fungal dysbiosis characterized by decreased diversity, an increased Ascomycota/Basidiomycota ratio, and an enrichment of pro-inflammatory, opportunistic pathogens that could alter the tumor microenvironment and immune response	Humans
	Fungi	*Candida albicans*, *C. tropicalis*, *C. dubliniensis*	Promotive	1. Immune modulation: induces pro-inflammatory pathways (IL-1β, Th17, cytokines IL-1A, IL-1B, IL-6, IL-8) 2. Cellular adhesion: downregulates genes involved in cell adhesion and epithelial barrier function, promoting metastasis 3. Trans-kingdom interactions: forms co-abundance groups with specific bacteria; negatively associated with *H. pylori* in stomach cancer	Humans/mice
	Fungi	*Candida albicans*, Ascomycota, Basidiomycota	Protective	Gut commensal fungi activate myeloid cells via the SYK-CARD9 axis, triggering NLRP3 inflammasome-dependent IL-18 production. IL-18 enhances epithelial barrier repair and promotes CD8^+^ T cell-derived IFN-γ to suppress tumor development	Humans/mice
	Fungi	*Candida tropicalis*	Promotive	Fungal dysbiosis resulting from CARD9 deficiency leads to an expansion of gut fungi, particularly *C. tropicalis*, which in turn promotes the accumulation and activation of MDSCs within the tumor microenvironment	Humans/mice
	Fungi	*Candida tropicalis*	Promotive	Enhances immunosuppressive function of MDSCs via the Dectin-3/Syk/PKM2/HIF-1α signaling axis, leading to aerobic glycolysis and increased production of immunosuppressive molecules (iNOS, COX2, NOX2, NO, ROS)	Humans/cell lines/mice
	Virus	*Myoviridae, Podoviridae, Siphoviridae, Drexlerviridae, Inoviridae*	Promotive	These phages could influence CRC by modulating bacterial host communities (dysbiosis) and/or through direct viral gene expression. The enrichment of viral functions related to fatty acid biosynthesis (e.g., stearate, oleate) could create a pro-carcinogenic metabolic environment	Humans
	Virus	*Herelleviridae*	Protective	Phages in this family typically infect Firmicutes and could contribute to intestinal barrier health. Its depletion in CRC suggests a potential loss of a protective function, possibly making the intestinal environment more permissive to tumor development	Humans
	Virus	*Microviridae, Podoviridae_crAss-like, Quimbyviridae*	Promotive	Indirect, via bacterial host modulation: Altering the gut bacterial community structure (dysbiosis). Specific enriched vOTUs are predicted to infect bacterial families such as *Bacteroidaceae*. Functional analysis indicated an enrichment of genes involved in methionine biosynthesis (K08968) in tubular adenomas-enriched viruses	Humans
	Virus	Torque teno virus (TTV) and other species from the *Anelloviridae* family (e.g., TTV-1, TTV-3, TTV-15, TTV-27)	Promotive	Potential mechanisms include: enrichment of viral adhesion genes; alteration of host cell metabolic pathways (glycerophospholipid metabolism, phosphatidylinositol signaling, primary bile acid biosynthesis); inhibition of host DNA repair mechanisms (base excision repair)	Humans
	Archaea	Enriched: *Natrinema* spp. J7-2 Depleted: Methanogenic archaea (e.g., various methanogens)	Context-dependent (Specific halophiles are promotive; methanogens could be protective)	Altered composition of archaea and disrupted interactions with bacteria in the gut microbiome are associated with colorectal carcinogenesis. The specific mechanistic role (driver or passenger) is not yet defined	Humans
	Archaea	Enriched: *Methanobrevibacter_A smithii*, *Methanobrevibacter_A sp002496065* Depleted: *Methanosphaera stadtmanae*, *Methanomassiliicoccus_A intestinalis*	Context-dependent (Specific methanogens are promotive; others are protective)	Altered archaeal composition and disrupted trans-kingdom interactions with bacteria are associated with CRC. Enriched methanogens (e.g., *M. smithii*) could promote a tumor-permissive environment, while depleted methanogens (e.g., *M. stadtmanae*) lose their protective interactions with butyrate-producing bacteria and exhibit reduced antagonism against pathogenic bacteria. Methanogenesis pathways are also dysregulated	Humans
Gastric cancer	Fungi	*Candida albicans*	Promotive	1. Fungal dysbiosis 2. Reduced diversity 3. Altered composition 4. Biomarker potential 5. Functional shift	Humans
	Fungi	*Solicoccozyma*	Protective	1. Associated with less advanced disease 2. Metabolic modulation	Humans
	Fungi	Enriched in GC: *Cutaneotrichosporon*, *Apiotrichum*, *Malassezia* Depleted in GC: *Rhodotorula*, *Rhizopus*, *Cystobasidium*	Promotive/Protective	Immune modulation: Positive correlation of GC-enriched fungi with pro-inflammatory (TNF-α, CXCL9/10/11) and IL-10 cytokines	Humans
	Virus	EBV	Promotive	EBV^+^ tumors are associated with distinct molecular alterations, including frequent PIK3CA (40%) and ARID1A (47%) mutations, and a characteristic immune response (high cytokine signature). They represent a unique molecular subtype with a specific tumorigenic profile (lack of CNV or hypermutation)	Humans
	Virus	EBV	Promotive	1. Induces host DNA hypermethylation (CIMP) 2. Promotes synergistic activation of PI3K-Akt and Wnt signaling pathways 3. Provides anti-apoptotic and proliferative signals via latent viral genes (e.g., EBNA1, LMP2A)	Humans/cell lines/mice
Esophageal cancer	Fungi	*Cladosporium cladosporioides*, *G. lagenaria*, *Fusarium* spp., *Candida albicans*, *Penicillium* spp.	Promotive	1. Autoreactive T cells permit fungal colonization and incite tissue injury and inflammation 2. Fungal infection promotes DNA damage 3. Inflammation and EGFR activity facilitate fungal persistence	Humans/cell lines/mice
	Virus	HPV types 16 and 18	Promotive	The specific mechanism is not detailed in this meta-analysis. However, it is widely known from other cancer contexts (such as cervical cancer) that the primary oncogenic mechanisms of HPV-16/18 involve the viral oncoproteins E6 and E7, which inactivate host tumor suppressor proteins p53 and pRb, respectively	Humans
Hepatocellular carcinoma	Fungi	*Aspergillus flavus*, *Aspergillus parasiticus*	Promotive	Production of aflatoxins (primarily Aflatoxin B1/AFB1), which are potent hepatocarcinogens	Humans
	Fungi	*Candida albicans*	Promotive	Intestinal colonization by *C. albicans* upregulates the expression of nucleotide-binding oligomerization domain-like receptor family pyrin domain containing 6 (NLRP6) in intestinal epithelial cells. This promotes HCC progression in a NLRP6-dependent manner	Humans/cell lines/mice
	Virus	HBV	Context-dependent (Promotive in the presence of environmental carcinogens)	HBV alone does not cause cancer. In the presence of environmental carcinogens (e.g., diethylnitrosamine/DEN), HBV alters the liver’s immune response. This leads to upregulation of the cytokine IL-33, which activates regulatory T cells (Tregs). These Tregs produce immunosuppressive cytokines (TGF-β, IL-10), creating an environment that suppresses anti-tumor CD8^+^ T cells and promotes liver carcinogenesis	Humans/mice
Pancreatic cancer	Fungi	*Malassezia globosa*, *Alternaria alternata*	Promotive	Intratumoral fungi activate the Dectin-1/Src/Syk/CARD9 signaling pathway in cancer cells, facilitating the secretion of IL-33. Secreted IL-33 recruits and activates pro-tumorigenic type 2 immune cells (TH2 cells and ILC2s) into the tumor microenvironment. These cells secrete cytokines such as IL-4 and IL-13, which promote tumor progression and metabolic reprogramming of cancer cells	Humans/cell lines/mice
	Virus	HBV	Promotive	The HBx enhances the malignancy of pancreatic cancer cells. It upregulates ErbB4 and TGF-α, leading to the activation of downstream oncogenic signaling pathways, primarily PI3K/AKT, as well as MAPK and ERK. This results in increased cell proliferation, migration, and induction of an EMT phenotype	Humans/cell lines
Oral cancer	Fungi	*Candida albicans*	Promotive	1. Production of carcinogenic acetaldehyde from ethanol metabolism 2. Enhanced biofilm formation (mass & metabolic activity) 3. Production of hydrolytic enzymes (e.g., phospholipase, proteinase) 4. Endogenous nitrosamine production	Humans
	Fungi	*Candida albicans*	Promotive	Indirectly via chronic inflammation and epithelial damage	Zebrafish/cell lines/mice

*Abbreviations*: *CARD* caspase recruitment domain-containing protein 9, *CIMP* CpG island methylator phenotype, *CNV* copy number variation, *CRC* colorectal cancer, *EBV* Epstein-Barr virus, *EMT* epithelial-mesenchymal transition, *GC* gastric cancer, *HBV* hepatitis B virus, *HBx* hepatitis B virus X protein, *HCC* hepatocellular carcinoma, *HPV* human papillomavirus, *MDSC* myeloid-derived suppressor cell, *vOTUs* viral operational taxonomic units

### Colorectal cancer

#### Fungi

Accumulating evidence implicates the gut mycobiome as a significant contributor to the pathogenesis of colorectal cancer (CRC), with fungal dysbiosis detectable from the pre-neoplastic stages of adenomas and polyps [59–62]. While the precise signature of this dysbiosis varies, it frequently manifests as a profound shift in the ratio of the two dominant phyla, Ascomycota and Basidiomycota, alongside a reduction in overall fungal diversity [59, 63]. This ecological restructuring is characterized by a marked enrichment of opportunistic and pro-inflammatory fungi. Genera including *Malassezia*, *Rhodotorula*, and *Acremonium*, as well as specific species such as *Aspergillus flavus*, are consistently reported to expand in CRC cohorts [59, 63]. A large-scale meta-analysis further solidified the existence of a CRC-associated mycobiome, identifying a core set of fungal species consistently enriched (e.g., *Aspergillus rambellii*, *Cordyceps* spp. RAO-2017, *Erysiphe pulchra*, *Moniliophthora perniciosa*, *Sphaerulina musiva*, and *Phytophthora capsici*) or depleted (e.g., *Aspergillus kawachii*) in patients’ feces [64]. Discrepancies in the reported direction of phyla-level shifts are likely attributable to methodological and cohort heterogeneities, yet the consensus firmly points to a loss of microbial equilibrium.

Critically, this dysbiosis is not confined to the fecal lumen but is recapitulated within the tumour tissue itself. Intratumoural fungal communities, heavily seeded from the gut, are predominantly composed of Ascomycota, particularly the class Saccharomycetes [65]. Detailed profiling of CRC tissues has revealed co-abundant networks of *Candida* species (including *C. albicans* and *C. tropicalis*) and *Saccharomyces*-associated clusters, suggesting synergistic ecological relationships among these fungi [27, 66–68]. The clinical relevance of these findings is underscored by studies linking specific fungi to disease progression. Most notably, the enrichment of *Malassezia restricta* within primary CRC tumours has been significantly associated with the development of distant metastasis, positioning the mycobiome as a potential modulator of clinical outcome [69]. Collectively, these findings point to a model where specific fungi, through direct interaction with the TME or by shaping the broader microbial community, actively influence CRC initiation and progression.

#### Viruses

The gut virome undergoes considerable restructuring in CRC, typically exhibiting increased diversity compared to healthy states [70, 71]. Early metagenomic sequencing established that the CRC-associated virome primarily consists of temperate bacteriophages, indicating a crucial role for virus-bacteria interactions in tumorigenesis [72]. Further research has identified specific bacteriophage families enriched in CRC, including *Myoviridae*, *Podoviridae*, *Siphoviridae*, *Drexlerviridae*, *Inoviridae*, and *Herelleviridae* [73]. Significantly, a 2025 study demonstrated that this dysbiosis commences at the premalignant stage of colorectal adenoma, characterized by a notable rise in viral families such as *Microviridae*, *Podoviridae_crAss-like*, and *Quimbyviridae* [74]. A comprehensive analysis identified 479 vOTUs with altered abundances, some of which are predicted to infect key bacterial families such as *Bacteroidaceae* and *Lachnospiraceae*, suggesting a mechanism whereby viral shifts could influence the bacterial microbiome towards a pro-oncogenic state [74]. Beyond bacteriophages, the mucosal virome of CRC tissues also exhibits alterations in eukaryotic viruses. The abundances of *Anelloviridae* and its representative species, Torque teno virus (TTV), are markedly elevated in both the colorectal mucosa and intestinal lamina propria of CRC patients [75]. Critically, functional analyses indicate that TTV sequences enriched within CRC tissues carry genes with potential pro-carcinogenic functions, suggesting a more direct role in tumour progression [75]. In addition, human polyomavirus 2, commonly known as the JC virus or John Cunningham virus (JCV) [76–78], and human cytomegalovirus (HCMV) [79–81] are frequently observed in CRCs. Thus, the CRC virome appears to contribute to oncogenesis through a dual mechanism: indirectly, via bacteriophage-mediated remodeling of the bacterial community, and directly, through the activity of eukaryotic viruses such as TTV, JCV and HCMV.

While early studies identified broad shifts in gut phage communities, a 2026 study by Damgaard et al. fundamentally shifted this view by linking specific prophages to the CRC driver *Bacteroides fragilis* [82]. Through pangenome-wide analysis of *B. fragilis* isolates obtained from patients with bacteremia, researchers identified two novel Caudoviricetes prophages, namely Bacteroides phage FU and ODE, which were significantly enriched in CRC-associated strains. These prophages integrate stably into distinct tRNA sites of the bacterial genome. Crucially, validation in fecal metagenomes from 877 individuals across multiple cohorts confirmed that CRC patients were twice as likely to carry detectable phage sequences (OR = 2.05). This discovery clarifies the paradox regarding how a prevalent commensal organism such as *B. fragilis* can play a role in oncogenesis. These particular prophages are likely responsible for inducing lysogenic conversion, which modifies the pathogenicity of host bacteria or introduces virulence-associated genes. Consequently, this research repositions the CRC virome from passive ecological shifts to active prophage-mediated bacterial reprogramming in dysbiosis. Moreover, strong cross-cohort association positions Bacteroides phages FU and ODE as promising non-invasive biomarkers for early CRC detection, with initial models showing 83.3% specificity.

In contrast to the complex ecological shifts observed in CRC, anal cancer exemplifies a paradigm of direct viral oncogenesis, predominantly driven by HPV [83]. The increasing incidence of anal cancer is mainly attributable to a rise in anal squamous cell carcinomas (SCCs), the histological subtype most closely linked with HPV infection, which constitutes approximately 88% of cases worldwide [84, 85]. The pathogenesis initiates with viral transmission via direct contact, whereby microabrasions in the epithelial surface facilitate access to and infection of basal keratinocytes [86]. This establishes a reservoir of infection that is essential for the subsequent development of malignancy.

#### Archaea

Emerging evidence suggests that the gut archaeome experiences significant restructuring during colorectal carcinogenesis. Distinct clustering of archaeal communities differentiates patients with colorectal adenoma and CRC from healthy individuals, indicating stage-specific alterations within this domain [87]. A multi-cohort study by Li et al. provided a definitive, large-scale validation of these shifts [88]. This study performed a meta-analysis of fecal metagenomes from 2,101 individuals across 11 independent cohorts spanning 7 countries (China, Austria, the United States, Italy, Japan, Germany, and France), comprising of 748 CRC patients, 471 adenoma patients, and 882 healthy controls (HCs). This large-scale and cross-continental design delineated robust, population-wide archaeal signatures associated with colorectal tumorigenesis [88]. A prominent aspect of this ecological shift appears to be a trade-off between methanogenic and halophilic archaea. Coker et al. [87] demonstrated that a depletion of methanogens alongside an enrichment of halophiles in patients with CRC, a trend exemplified by the progressive increase of *Natrinema* spp. J7-2 from health to adenoma to carcinoma, which implies an association between a saline colonic environment and tumour development.

However, the dynamics of methanogens are intricate and cannot be solely characterized by loss. Data indicated a gradual enrichment of the methanogenic phylum Methanobacteriota throughout the healthy-adenoma-CRC sequence [88]. Resolution at the species level from this multi-cohort meta-analysis uncovered a nuanced perspective: while certain species such as *Methanobrevibacter_A smithii* (consistently the most prevalent and abundant archaeon, enriched in CRC across multiple cohorts) and *Methanobrevibacter_A sp002496065* were enriched in CRC, others, including *Methanosphaera stadtmanae* and *Methanomassiliicoccus_A intestinalis*, were persistently depleted across multiple cohorts. Crucially, the study identified a subset of archaea exhibiting a clear, progressive decrease following the HC-adenoma-CRC sequence. These stage-specific species included *M. stadtmanae*, *Methanobrevibacter_A sp900314695*, and *Methanocorpusculum sp001940805*, underscoring their potential role as ecological markers of disease progression from precancerous lesions to invasive cancer [88]. This heterogeneity highlights the likelihood that archaeal contributions to CRC are species- and function-specific, transcending mere abundance trends to delineate a functionally altered archaeome in CRC. Furthermore, the study revealed that CRC-depleted methanogenic archaea, such as *M. intestinalis* and *M. stadtmanae*, exhibited enhanced co-occurrence with short-chain fatty acid (SCFA)-producing bacteria *Roseburia intestinalis* and *Butyribacter hominis*. This suggests that the loss of these archaea may disrupt syntrophic networks that are crucial for colonic homeostasis [88].

### Hepatocellular carcinoma

#### Fungi

The gut mycobiome is increasingly recognized as an important factor in hepatocellular carcinoma (HCC) development through the gut-liver axis. As presented in a 2025 conference report, Zhou et al. [89] showed that a high-fat/high-cholesterol diet causes progressive fungal imbalance in mice, marked by a significant increase in *Rhizopus arrhizus*, which was enough to promote metabolic dysfunction-associated steatotic liver disease-related hepatocellular carcinoma (MASLD-HCC). This is supported by human data, where fecal analyses reveal consistent intestinal fungal imbalance in HCC, with an increase in opportunistic pathogens such as *Malassezia* and *Candida* species compared to HCs or cirrhotic patients [90, 91]. The presence of *C. albicans* specifically correlates with more advanced tumour stages [90]. Beyond the gut, the fungal community within the HCC tumour mass itself exhibits a distinct and pathogenic composition. Shen et al. [92] demonstrated that the fungal community infiltrating HCC tumours is fundamentally different from that in adjacent normal tissue (NAT), with a marked and specific enrichment of *Malassezia* species within the tumours. This finding situates pro-tumorigenic fungi at the very site of malignancy, suggesting potential local mechanisms of action. Complementing the role of living commensals, carcinogenic mycotoxins (e.g., aflatoxins from *Aspergillus* species) represent some of the most potent chemical hepatocarcinogens, completing the picture of fungi as multifactorial contributors to HCC through both direct and indirect mechanisms (Section “Microbial-derived metabolites and toxins”) [93–95].

#### Viruses

In contrast to the emerging role of fungi, the etiology of HCC is predominantly driven by chronic infection with hepatotropic viruses. Chronic hepatitis B virus (HBV) infection constitutes the primary global risk factor, accounting for approximately 50% of HCC cases [96]. Individuals with HBV-related cirrhosis face a markedly increased risk, with a 31-fold higher likelihood of developing HCC and a 44-fold higher mortality rate compared to non-cirrhotic individuals. Additional risk factors include male gender, advanced age, and specific viral genotypes [97, 98]. Hepatitis C virus (HCV) infection is another significant contributor, responsible for approximately 30% of HCC cases worldwide [99]. HCV possesses a particularly high oncogenic potential, as the annual incidence of de novo HCC in patients with HCV-related cirrhosis is up to twice that observed in cirrhosis from other etiologies, and it can occur even in the absence of cirrhosis [100–102]. Furthermore, co-infection with hepatitis D virus (HDV) in HBV carriers further amplifies the risk, thereby establishing chronic viral hepatitis as the most critical causative factor for HCC globally [103].

### Gastric cancer

#### Fungi

The gastric mycobiome is gaining recognition as a potential contributor to gastric carcinogenesis, with specific fungi implicated in disease progression and patient outcomes. Dohlman et al. identified a significant enrichment of *Candida* species, particularly *C. tropicalis*, within tumour tissues compared to adjacent normal mucosa, an association linked to decreased patient survival [27]. This fungal dysbiosis extends beyond *Candida*, characterized by a broader increase in opportunistic fungi (e.g., *Cutaneotrichosporon* and *Malassezia*) and a loss of beneficial taxa such as *Rhizopus* and *Rhodotorula* [104]. Enrichment of *C. albicans*, for example, correlates with shifts in a co-abundance network involving *Fusicolla acetilerea*, *Arcopilus aureus*, and *Fusicolla aquaeductuum*, whereas other species such as *Candida glabrata*, *Aspergillus montevidensis*, *Saitozyma podzolica* and *Penicillium arenicola* are depleted [105].

Nevertheless, the landscape remains intricate and subject to controversy. Contrary to the findings previously mentioned, Zhang et al. [106] did not observe a significant enrichment of *Candida* in tumours. Instead, they identified *Solicoccozyma* as a differentially abundant genus, with higher levels detected in early-stage disease. Additionally, a prospective cohort study determined that there was no correlation between *Candida*-related oral lesions and gastric cancer (GC) risk [107]. These inconsistent findings are likely attributable to methodological, cohort, and regional heterogeneities, emphasizing the necessity for larger, standardized studies to establish reliable fungal signatures and to elucidate their specific mechanistic roles in GC.

#### Viruses

Compared to the emerging and intricate role of fungi, the involvement of specific oncogenic viruses in GC is more definitively established. The EBV is identified as the second most prevalent infectious agent, following *Helicobacter pylori*, implicated in gastric oncogenesis [43]. Its presence within GC cells has been independently confirmed [108–111]. A comprehensive meta-analysis involving over 20,000 patients verified that EBV is detected in approximately 8.8% of GC cases and is associated with an over 18-fold increased risk of the disease, thereby confirming its significance as a key oncogenic driver in a distinct subset of patients [112].

The evidence regarding HPV is less consistent but indicates a possible association with considerable geographic variation. A pooled analysis revealed an overall HPV prevalence of 28.0% among GC patients, with an odds ratio of 7.4 for GC risk, although there was substantial heterogeneity [113]. The prevalence was significantly higher in Chinese cohorts (31%) compared to non-Chinese regions (9%), with HPV16 being the predominant genotype [113]. While these findings are compelling, they necessitate further validation, as other reports have suggested only an isolated role for HPV in GC [114, 115]. Consequently, although EBV is a well-established etiological agent, the contribution of HPV appears to be dependent on specific contexts and requires additional investigation.

### Pancreatic cancer

Pancreatic cancer, now the third leading cause of cancer-related mortality, exhibits persistently rising death rates, underscoring the urgent need to elucidate novel pathogenic drivers [1]. Emerging evidence implicates the gut microbiome as a key contributor, with specific microbes demonstrating the capacity to translocate to the pancreas and foster the tumorigenesis and progression of pancreatic ductal adenocarcinoma (PDAC) [28, 29, 116]. A pivotal study by Aykut et al. [28] illuminated the fungal mycobiome in this process, demonstrating its translocation from the gut lumen to the pancreas via the sphincter of Oddi. This migration resulted in an enrichment of the pancreatic fungal population by over 3,000-fold in PDAC compared to normal tissue. Their analysis identified Ascomycota and Basidiomycota as the dominant phyla in both human and murine guts and PDAC tumours, with a notable specific enrichment of the genus *Malassezia* in human PDAC tissues [28].

However, the reproducibility of these specific fungal signatures has been questioned. A re-analysis by Fletcher et al. [117] of the same sequencing data failed to corroborate the reported differences in human pancreatic or fecal samples. This discrepancy highlights a critical challenge that extends far beyond this particular study and is characteristic of low-biomass microbiome research. Tissues such as the pancreas, which are not directly exposed to gut microbiome, possess exceedingly low microbial loads. As a result, they are susceptible to contamination from laboratory reagents, environmental sources, and the host’s circulating microbial DNA [118]. Additionally, the limited initial quantity of microbial DNA intensifies amplification biases during library preparation, potentially distorting taxonomic representation. These studies emphasize the necessity for standardized and rigorous protocols, including implementation of comprehensive negative controls, spike-in standards, and computational decontamination algorithms, to ensure the generation of robust and reproducible results within this emerging field [117, 119].

Beyond fungi, viral pathogens have also been implicated in PDAC. A large prospective cohort study of 496,732 Chinese adults revealed that hepatitis B virus surface antigen (HBsAg) seropositivity was associated with an increased risk of pancreatic cancer [120]. This population-level association is supported by tissue-based evidence confirming the presence of HBV within pancreatic cancer cells [120]. Further strengthening this link, in vitro functional studies demonstrate that the HBV X protein (HBx) exerts direct oncogenic effects by promoting the proliferation and migration of PDAC cells [121], collectively suggesting a potential role for HBV in pancreatic oncogenesis [122, 123].

### Esophageal cancer/oral cancer

Emerging evidence implicates the fungal microbiome, particularly *C. albicans*, in the pathogenesis of both esophageal cancer (EC) and oral cancer (OC) [124–126]. The potential oncogenic role of *C. albicans* is supported by experimental models demonstrating that its depletion inhibits, while its administration accelerates, the development of esophageal squamous cell carcinoma (ESCC) [127]. This could be mediated, in part, through the induction of T-lymphocyte dysfunction in infected patients. Beyond *Candida*, microbiome analyses have identified other fungal genera with enriched abundance in these niches, such as *Rhodotorula*, *Geotrichum*, and *Pneumocystis* in OC [128], and *Malassezia*, *Saccharomyces*, *Aspergillus*, and *Cyberlindnera* in the salivary microbiome [129], although their functional roles require further elucidation.

In contrast to the emerging association with fungi, the role of high-risk HPV as a primary etiological factor in oropharyngeal squamous cell carcinoma (OPSCC) is well established. The evidence for the increasing incidence of HPV-associated OPSCC derives from study that detects viral oncogene activity (e.g., E6/E7 transcripts) or the surrogate marker p16-INK4a, which is consistently overexpressed in these tumours [130]. Globally, the proportion of HPV- and p16-positive OPSCCs is on the rise [131–134]. This trend is strongly supported by divergent epidemiological patterns: as the incidence of lung cancer declines in many countries, the incidence of OPSCC increases, indicating that HPV infection, rather than tobacco, serves as the primary causative factor [135]. Additionally, an increasing average age at diagnosis suggests a significant cohort effect [132, 136–138]. The burden of HPV-positive OPSCC is most significant in high-income countries in North America and Europe [84, 139]. Beyond the oropharynx, the oncogenic potential of HPV could extend to the esophagus. Although esophageal squamous papillomas are typically benign, concerns exist regarding their recurrence and malignant potential, especially when linked to high-risk HPV genotypes [140]. A growing, though not yet definitive, body of evidence has identified HPV-16 and −18 not only in benign lesions but also in ESCC specimens [140, 141], and meta-analyses have confirmed a statistically significant correlation between HPV infection and ESCC risk [142, 143]. Thus, HPV is positioned as a microbial factor of interest in esophageal carcinogenesis alongside *Candida*.

## Causality and molecular mechanisms

Having established the correlative links between non-bacterial microbes and various GI cancers, we now examine the underlying causality and molecular mechanisms. While each cancer type possesses a unique tissue environment, several shared mechanistic themes have emerged from the literature reviewed in the previous section. A recurring hallmark is the activation of innate immune SYK-CARD9 axis by fungal pathogen-associated molecular patterns (PAMPs), which drives tumour-promoting inflammation and immune suppression in both colorectal and pancreatic cancers. On the other hand, the induction of a pathogenic Th17/IL-17 response by *Candida* species is implicated in the progression of both esophageal and colorectal malignancies. However, it is critical to distinguish between findings derived from association studies and those supported by functional or causal evidence from in vitro and in vivo models. This section will prioritize mechanistic insights, highlighting the direct and indirect pathways by which viruses, fungi, and archaea hijack host cellular processes to promote oncogenesis. Key unresolved questions remain, such as the molecular triggers that transform a commensal fungus into a pathogenic state and the precise nature of the reciprocal signaling networks that regulate cross-kingdom interactions within the TME. These mechanistic studies offer insights into how non-bacterial microbiome functions as an integral regulator of GI cancers.

### Chronic inflammation

Chronic inflammation is a hallmark of cancer and an established risk factor of cancer initiation and progression, especially within the GI tract [144, 145]. Commensal and opportunistic fungi are significant regulators of the host immune response, capable of inducing a pro-tumorigenic inflammatory environment [27, 68, 146, 147]. Innate immune cells, including macrophages, dendritic cells (DCs), and natural killer T (NKT) cells, recognize fungal PAMPs, such as β-glucans, chitin, and mannans, through an array of pattern recognition receptors (PRRs) [148]. Notably, C-type lectin receptors (CLRs), such as Dectin-1, −2, −3, and Mincle, play a critical role in fungal detection [149]. Engagement of these receptors typically initiates a signaling cascade involving spleen tyrosine kinase (SYK) and the adaptor protein CARD9, which is predominantly expressed in myeloid cells [150]. This SYK-CARD9 signaling axis regulates antifungal immunity by facilitating immune cell infiltration, activation, and the secretion of pivotal pro-inflammatory cytokines (e.g., IL-1β, IL-6, IL-23, TNF-α, IFN-γ) via activation of NF-κB, MAPK, and STAT signaling pathways [151–156]. Critically, the functional outcome of this fungal sensing is context-dependent. While chronic, dysregulated activation is tumour-promoting, the same SYK-CARD9 pathway can, in certain settings, induce IL-18 and confer protection against colitis-associated colon cancer by bolstering anti-tumour T-cell immunity [157]. The specificity of this response is further refined by CLR heterodimerization, as exemplified by Dectin-3 partnering with Dectin-2 to broaden ligand recognition [158, 159]. The pro-tumorigenic potential of these pathways is mechanistically demonstrated in oncological models. For instance, the pancreatic tumour mycobiome drives tumour progression via Dectin-1-mediated Src-Syk-CARD9 signaling, which induces IL-33 release and subsequently polarizes a pro-tumour Th2/ILC2 response [29]. Similarly, *C. albicans* can accelerate EC progression via an IL-17A-dependent mechanism in macrophages [160], and Th17 cells primed by *C. albicans* can cross-react with other fungi, exacerbating intestinal inflammation [161]. Collectively, these findings delineate a mechanism by which fungi exploit innate immune networks to foster a microenvironment conducive to carcinogenesis.

Emerging evidence positions bacteriophages as an additional crucial element of the microbiota capable of mediating both direct and indirect immunomodulatory effects. Phage nucleic acids (ssDNA, dsDNA) function as ligands for various PRRs, including endosomal TLR9 and cytosolic sensors such as cGAS and AIM2, thereby activating MyD88- or STING-dependent pathways that lead to cytokine secretion (e.g., IFN-α, IL-6, IL-12) [162, 163]. Filamentous phages, exemplified by M13, have the capacity to persist within mucosal tissues and, upon internalization by epithelial and antigen-presenting cells, influence downstream immune signaling pathways and antigen presentation processes [164]. The ensuing immune response is notably pleiotropic. Bacteriophages can exert both pro-inflammatory effects, such as promoting Th1 responses and TNF production, and anti-inflammatory effects, including the amelioration of inflammation in inflammatory bowel disease (IBD) models [163, 165, 166]. This duality is dictated by factors including phage type, structure, and the local host environment [165]. Furthermore, phages indirectly modulate immunity through bacterial lysis, which releases immunostimulatory bacterial PAMPs (e.g., LPS) that can perpetuate inflammation via TLR signaling [162]. Thus, both the mycobiome and the virome, through their intricate and often paradoxical interactions with the host immune system, emerge as critical regulators of tumorigenesis. Understanding the balance between their protective and pathogenic roles, and how this balance is disrupted in disease, represents a frontier in cancer research.

### Immunomodulation

#### Immunosurveillance

Immunosurveillance exerts significant selective pressure on tumour cells, driving the evolution of mechanisms to evade immune destruction [167]. Fungal dysbiosis serves as a significant trigger for the recruitment and functional polarization of myeloid-derived suppressor cells (MDSCs), a heterogeneous group of immature myeloid cells that inhibit T cell activity through the production of reactive oxygen species (ROS), nitric oxide (NO), arginase, and other mediators [168, 169]. A notable example is observed in the context of CARD9 deficiency, where overgrowth of commensal fungi (e.g., *C. tropicalis*) induces the accumulation of MDSCs within the colorectal TME, thereby accelerating tumour progression by suppressing CD8^+^ and CD4^+^ T cell responses [170]. Mechanistically, *C. tropicalis* activates the Syk-PKM2-HIF-1α signaling axis in MDSCs, initiating a robust glycolytic pathway crucial for their immunosuppressive function [171–173]. This creates a direct connection between fungal sensing and metabolic reprogramming involved in immune evasion. Moreover, *C. tropicalis* can also promote the secretion of IL-1β from MDSCs via the NLRP3 inflammasome, which in a positive feedback loop, further enhances their immunosuppressive capacity [174].

Beyond MDSCs, viruses can exploit regulatory T cells (Tregs) to establish a state of local immune tolerance. In the context of HBV-induced hepatocarcinogenesis, viral infection results in the upregulation of IL-33. This cytokine subsequently activates Tregs, which release immunosuppressive cytokines such as TGF-β and IL-10, thereby suppressing anti-tumour CD8^+^ T cell activity and facilitating tumour progression [44]. Another sophisticated immune evasion strategy employed by oncogenic viruses is the concerted upregulation of immune checkpoint molecules. Epstein-Barr virus-associated gastric cancer (EBVaGC) provides a clear paradigm, characterized by the overexpression of multiple inhibitory receptors and enzymes [110, 175]. A key player is indoleamine 2,3-dioxygenase 1 (IDO1), which inhibits T cell function by depleting tryptophan and accumulating kynurenine metabolites in the TME, thereby promoting tumour persistence and metastasis [109, 176]. This is compounded by the upregulation of surface checkpoint regulators such as PD-1, LAG3, CTLA4, and TIGIT, collectively erecting a formidable barrier to effective anti-tumour immunity [110, 175].

#### Complement system

The complement system serves as a vital connection between innate and adaptive immunity, and its activation products can significantly influence tumour dynamics [177]. Its function in antifungal defense is well-documented, as evidenced by the increased susceptibility to *Candida* infections in mice deficient in essential complement components such as C3 or C5 [178, 179]. This protective mechanism is primarily mediated by the anaphylatoxin C5a, which signals through its receptor, C5aR, to recruit and activate macrophages for fungal eradication via nonoxidative pathways [180]. While *Candida* can activate the classical, alternative, and lectin pathways, evidence indicates that the Mannose-Binding Lectin (MBL) pathway is particularly crucial for initiating opsonophagocytosis by neutrophils [181, 182]. The recognition of fungal cell wall glycans by MBL not only initiates complement activation but has also been linked to the promotion of carcinogenesis, thereby demonstrating the complex role of the system in disease processes [28]. To counteract this potent host defense, *Candida* and other fungi have developed sophisticated evasion strategies [183–185]. These strategies encompass molecular mimicry to evade initial recognition and, more actively, the degradation of complement components through the secretion of proteases [186–188]. A principal approach involves the strategic hijacking of host complement regulators, such as Factor H [189, 190]. By recruiting these soluble regulators to its cell surface, the fungus establishes a microenvironment of localized complement inhibition, thereby effectively deactivating the immune response at the site of contact [191].

### Microbial-derived metabolites and toxins

In addition to shaping immune responses, gut microorganisms fuel tumorigenesis through a varied array of metabolites and toxins. These substances can induce genetic instability, reprogram host cell metabolism, and compromise tissue integrity, thereby establishing microbial metabolism as a fundamental component of the tumour-promoting microenvironment [192, 193]. Fungi are prolific producers of bioactive metabolites with direct oncogenic properties. A prime example is the recent identification of kynurenic acid (KYNA) as a key oncometabolite enriched by *R. arrhizus*. KYNA acts as a potent driver of proliferation in MASLD-HCC models, simultaneously suppressing cell cycle arrest and apoptosis while fueling the growth of murine organoids [89]. This highlights a direct pathway through which fungal dysbiosis can orchestrate pro-tumorigenic phenotypes. The opportunistic pathogen *C. albicans* employs a multi-faceted offensive strategy [126, 185, 194]. Its virulence is partly attributable to a cytolytic peptide toxin that directly permeabilizes epithelial membranes. This breach triggers uncontrolled calcium influx and initiates a danger-response signaling pathway, culminating in chronic immune activation and tissue damage [195]. Furthermore, *C. albicans* functions as a metabolic converter, producing specific hydrolytic enzymes that transform dietary alcohol into carcinogenic acetaldehyde. The accumulation of acetaldehyde-DNA adducts represents a well-established mechanism linking fungal colonization to oral carcinogenesis, particularly in the context of chronic alcohol consumption [196–200]. The carcinogenic portfolio of *Candida* extends to the production of nitrosamines, such as N-nitrosobenzylmethylamine, which are potent inducers of genetic instability [201, 202]. Environmental fungi serve as equally potent agents. Aflatoxins, especially Aflatoxin B1 produced by *Aspergillus* species, rank among the most potent carcinogens known to humans and are etiologically associated with HCC. Their genotoxic effects primarily originate from the induction of significant oxidative stress, which results in direct oxidative damage to DNA and the formation of lipid peroxidation by-products that further degrade cellular integrity [203–207]. Additionally, other fungal-derived genotoxins, such as fumonisin and patulin, exemplify this concerning biological phenomenon [208, 209]. The tumour-promoting influence of metabolites extends beyond direct genotoxicity to systemic metabolic reprogramming. Multi-omics approaches have demonstrated that colonization of *C. albicans* in patients with HCC significantly alters the host plasma metabolome. These modifications, characterized by alterations in metabolites such as L-carnitine and disruptions in essential pathways such as the citrate cycle, indicate a substantial manipulation of host energy metabolism [91]. Similarly, tumour-resident *Malassezia* species can facilitate a pro-tumour environment by inhibiting bile acid synthesis through the downregulation of key enzymes CYP7A1 and CYP27A1 [92]. Besides fungi, viral pathogens can also cause cellular damage through toxin-like mechanisms. For example, SARS-CoV-2 infects and replicates within GI tissues, utilizing high levels of ACE2 receptor expression. The subsequent viral replication and the effects of viral toxins directly contribute to cellular injury, thereby broadening the understanding of toxin-mediated microbial pathogenesis in the GI tract [210, 211].

### Genotoxicity and genomic instability

Apart from the production of metabolites, commensal and pathogenic microbes directly hijack and manipulate core cellular signaling pathways, thereby promoting a pro-tumorigenic state through the induction of metabolic stress, genomic instability, and epigenetic dysregulation [212, 213]. Fungi, particularly *C. albicans*, are potent activators of oncogenic signaling cascades. It can trigger the MAPK and NF-κB pathways, which in turn modulate the host’s hypoxia response by stabilizing and activating HIF-1α, a regulator that activates cellular responses to low-oxygen conditions and angiogenesis [67, 214–216]. This signaling synergy promotes a microenvironment conducive to cancer progression. Furthermore, pan-cancer analyses link a high abundance of *Candida* to the downregulation of cellular adhesion genes, suggesting a role in compromising the intestinal barrier to facilitate CRC progression [27]. The tumour-promoting influence of *C. albicans* extends to oral squamous cell carcinoma (OSCC), where it upregulates oncogenes, induces matrix metalloproteinases, and promotes the production of onco-metabolites, thereby driving the entire spectrum of carcinogenesis from initiation to metastasis [217].

**Fig. 2:**
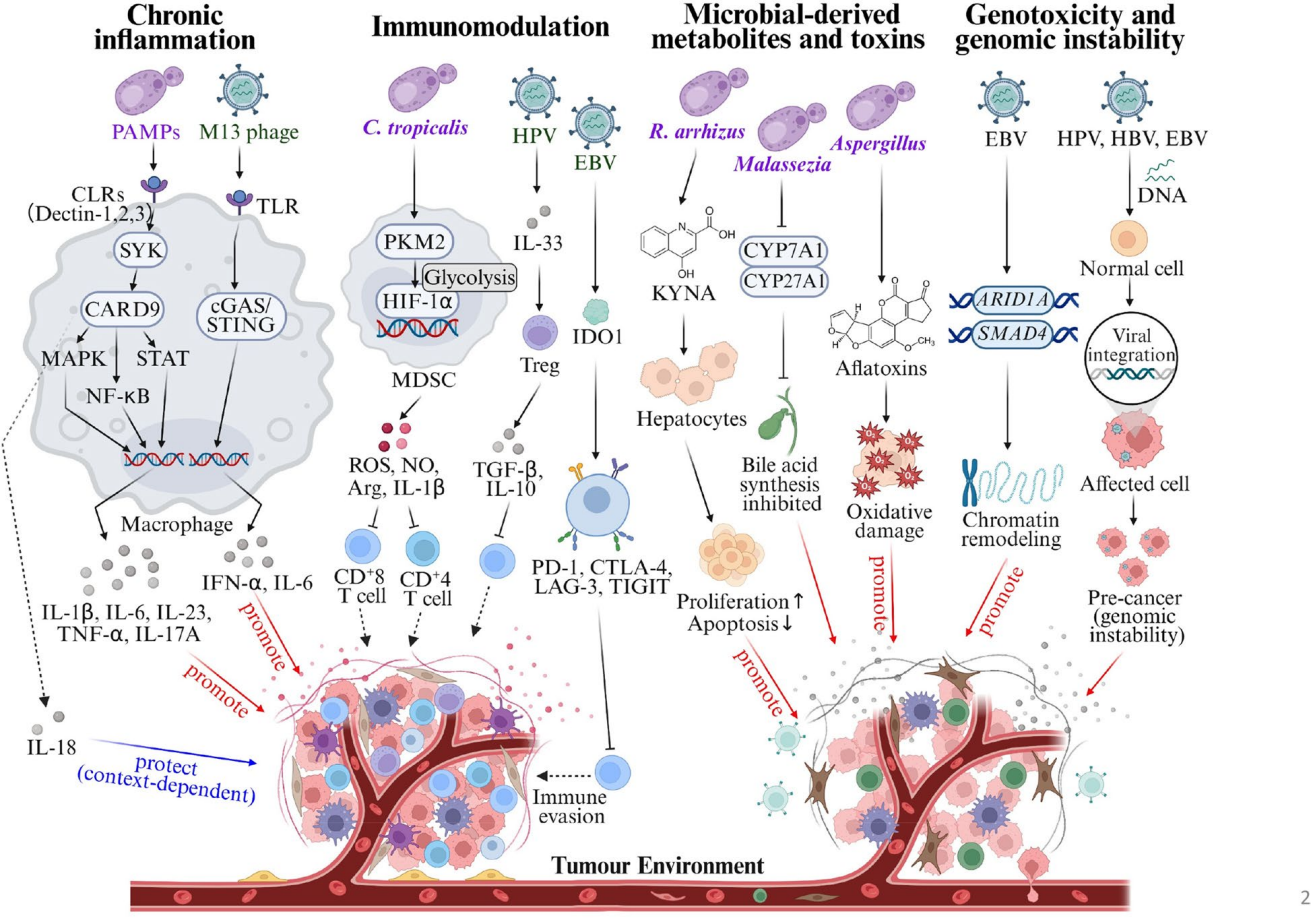
Multifaceted mechanisms of the non-bacterial microbiota in gastrointestinal tumorigenesis. CLR, C-type lectin receptors; KYNA, kynurenic acid; PAMPs, pathogen-associated molecular patterns; SYK, spleen tyrosine kinase; Treg, regulatory T cell

Oncogenic viruses induce tumorigenesis via both direct and indirect mechanisms, leading to significant genetic and epigenetic modifications. EBVaGC demonstrates a distinctive mutational landscape. A characteristic feature is the high frequency of somatic mutations in *PIK3CA*, resulting in the constant activation of the pro-survival Akt pathway [218–221]. Concurrent mutations often impact key genes involved in Wnt and Notch signaling pathways (*CTNNB1*, *NOTCH1*), cell cycle regulation, and chromatin remodeling (*ARID1A*, *SMAD4*), thereby establishing a permissive environment for cellular transformation [222]. Additionally, EBV encodes a range of viral microRNAs (miRNAs) that directly suppress host pro-apoptotic genes and modulate the expression of both viral and cellular genes to enhance cell survival [223, 224]. Spanning this mechanistic spectrum, HBV employs a dual strategy. Its direct oncogenic potential is realized through random integration into the host genome, which can induce chromosomal translocations and amplify genetic instability, thereby prolonging the expression of viral oncogenes [225, 226]. The multifunctional HBx protein is a central orchestrator of this process. It is essential for the transcriptional maintenance of viral covalently closed circular DNA (cccDNA) and dysregulates a network of host signaling pathways, including Wnt/β-catenin, PI3K/AKT, and STAT3, to drive proliferation [121, 227, 228]. HBx also induces extensive epigenetic modifications, such as DNA and histone methylation, to silence tumour suppressor genes [229]. A paradigm-shifting study from 2025 refines our understanding of HBV’s role, demonstrating that the virus alone could be insufficient to trigger inflammation or cancer. Instead, HBV functionally reprograms the chronic inflammation induced by chemical carcinogens such as diethylnitrosamine (DEN), thereby coopting the host’s immune response to dramatically promote liver carcinogenesis [44]. Moreover, JCV has been implicated in CRC through mechanisms that introduce widespread genetic and epigenetic instability [230] (Fig. 2).

### Cross-kingdom interactions and ecological dysbiosis

**Fig. 3:**
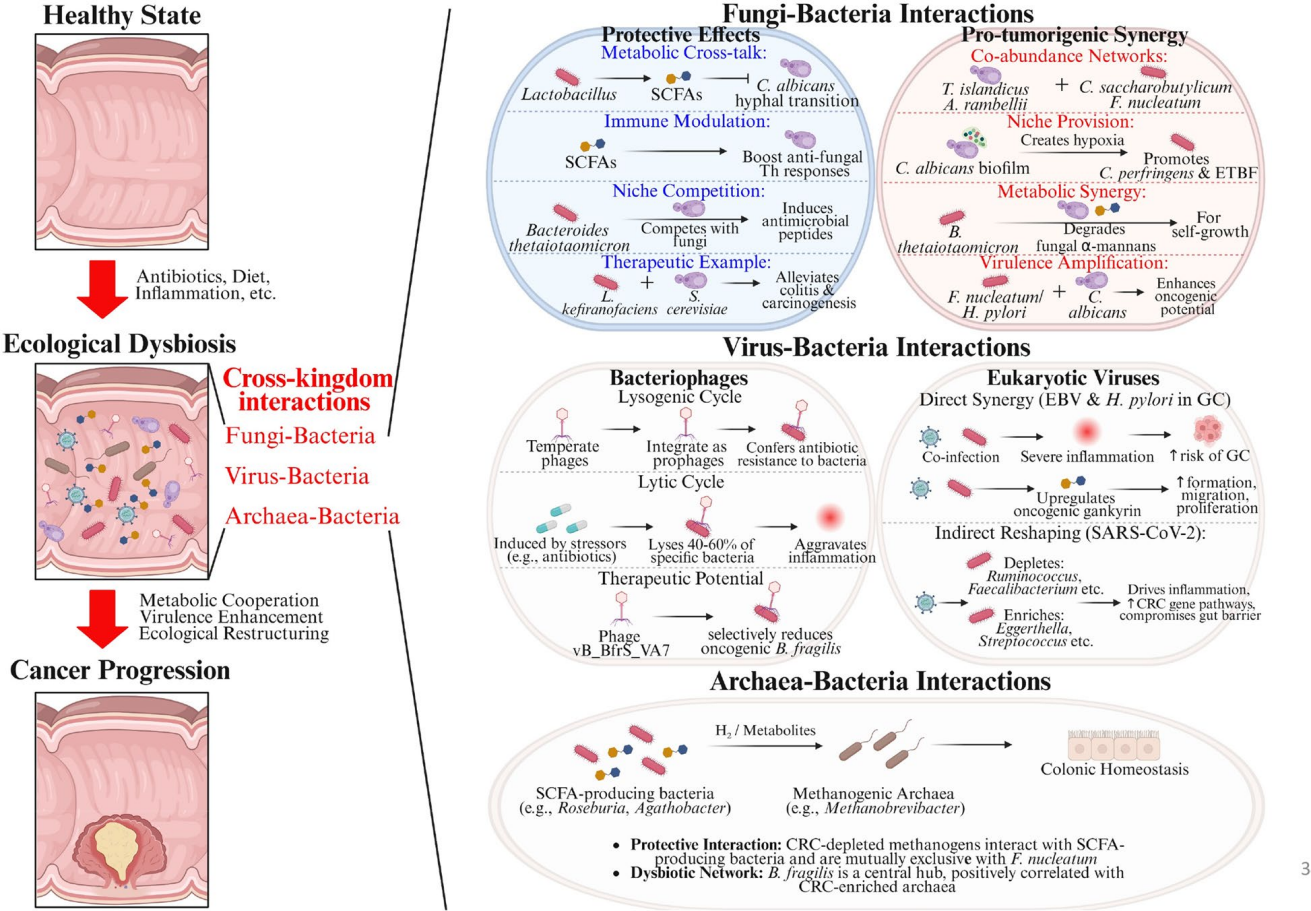
A Cross-Kingdom consortium of fungi, viruses, archaea, and bacteria in gastrointestinal carcinogenesis. CRC, colorectal cancer; ETBF, enterotoxigenic *Bacteroides fragilis*; GC, gastric cancer; SCFA, short-chain fatty acid; Th, T helper

Carcinogenesis within the GI tract is propelled by a complex cross-kingdom ecological system, rather than solely by isolated microbes. Fungi, viruses, and archaea participate in intricate interactions with bacteria and the host, forming synergistic consortia that promote tumour development. These networks, characterized by metabolic cooperation, virulence enhancement, and ecological restructuring, collaboratively establish a dysbiotic microenvironment that is integral to the advancement of cancer (Fig. 3 and Table 2).

**Table 2:** Transkingdom crosstalk in the gut microbiome: Implications for gastrointestinal carcinogenesis

Interacting kingdoms	Specific microbes/components	Mechanism of interaction	Functional outcome	Potential role in GI cancer
Fungi-Bacteria	Fungus: *Candida albicans* Bacteria: *Lactobacillus* spp. (e.g., *L. rhamnosus*, *L. reuteri)*	1. Secretion of 1-ABC: This small molecule inhibits the *C. albicans* yeast-to-hyphae transition by targeting and inhibiting the fungal kinase Yak1, a member of the DYRK1 family 2. Genetic bypass: Mutations in fungal transcription factor Rob1 (downstream of Yak1) or phosphatase Oca6 (upstream of Yak1) can restore filamentation in the presence of 1-ABC	1. Inhibition of virulence: 1-ABC and its more stable analog, 1-ECBC, potently block *C. albicans* filamentation and biofilm formation in response to diverse cues 2. Maintenance of commensalism: By preventing a key virulence trait (hyphal growth), this interaction helps maintain *C. albicans* in a commensal, non-pathogenic state	While not directly tested in GI cancer models, the mechanism is highly relevant. *C. albicans* hyphae are associated with tissue damage, inflammation, and potential procarcinogenic effects. By suppressing hyphal morphogenesis, *Lactobacillus*-secreted 1-ABC could theoretically reduce fungus-driven inflammation and epithelial damage in the GI tract, potentially lowering a risk factor associated with carcinogenesis
	Fungus: *Saccharomyces cerevisiae* JKSP39 isolated from Tibetan kefir grain Bacterium: *Lactobacillus kefiranofaciens* JKSP109	1. Co-administration modulates gut microbiota 2. Enhances intestinal barrier 3. Increases SCFA production 4. Reduces inflammation 5. Promotes apoptosis	1. Alleviates colitis and carcinogenesis 2. Improves gut health	Potential therapeutic or preventive agent
	Fungus: *Candida albicans* Bacteria: *Clostridial Firmicutes* (Clusters IV, XIVa) and *Bacteroidetes* (e.g., *Bacteroides thetaiotaomicron*, *Blautia producta*)	Commensal anaerobes, through their presence or specific components (e.g., from *B. thetaiotaomicron*), activate the host transcription factor HIF-1α in colonic epithelial cells. This activation leads to the upregulation and expression of the antimicrobial peptide LL-37 (CRAMP in mice). LL-37 possesses direct anti-*Candida* activity, inhibiting its growth and adhesion	1. Inhibition of *C. albicans* GI colonization: The activation of the HIF-1α/LL-37 axis by commensal bacteria is a key determinant of colonization resistance, preventing *C. albicans* from establishing a persistent population in the gut 2. Protection from invasive disease: Pharmacologic activation of this pathway with the HIF-1α agonist L-mimosine significantly reduced *C. albicans* colonization and decreased mortality from subsequent disseminated infection	While not directly tested in this study, chronic colonization by *C. albicans* has been associated with GI cancers. By preventing persistent colonization and the associated chronic inflammation and potential carcinogenic metabolite production (e.g., acetaldehyde) by *C. albicans*, the commensal bacteria-mediated activation of HIF-1α/LL-37 could indirectly reduce a potential risk factor for GI oncogenesis
	Fungal component: Yeast cell wall α-mannan (from *Saccharomyces cerevisiae*, *Candida albicans*, *Schizosaccharomyces pombe*) Bacteria: *Bacteroides thetaiotaomicron* (primary focus), also *Bacteroides ovatus* and specific *Parabacteroides* species	1. ‘Selfish’ degradation by polysaccharide utilization loci - Limited surface cleavage - Periplasmic depolymerization 2. Distinct from host glycan processing 3. Metabolic isolation (‘Selfish’)	1. Bacterial growth and colonization advantage 2. Evolutionary adaptation 3. Niche specialization	While not directly tested in GI cancer models, it notes that yeast mannans are implicated in the immunopathology of Crohn's disease (a risk factor for CRC)
	Fungus: *Candida albicans* Bacterium:* Helicobacter pylori*	Endosymbiosis: *H. pylori* is internalized into the vacuoles of *C. albicans* yeast cells, forming moving “bacteria-like bodies” (BLBs)	1. Protection from stress 2. Antibiotic resistance 3. Transmission and colonization	While not directly tested in this study, the described interaction provides a mechanism for persistent *H. pylori* infection despite antibiotic treatment. This protection and facilitation of persistence could indirectly promote carcinogenesis by allowing ongoing inflammation and damage
Virus-Bacteria	Virus: lytic phage, vB_BfrS_VA7 (Phage VA7) Bacterium: *Bacteroides fragilis*	Lytic infection: Phage VA7 specifically infects and lyses *B. fragilis* cells. It adheres to the bacterial surface, injects its genetic material, replicates within the bacterium, and causes bacterial cell lysis, releasing new phage particles	Effective and selective reduction of *B. fragilis* load in the mouse gut and within colon tumors. Does not affect other bacteria such as *E. coli*. The elimination of *B. fragilis* reverses its pro-chemo-resistance effects	Therapeutic agent to restore chemosensitivity. Phage VA7, by precisely targeting *B. fragilis*, abolishes the bacteria-induced chemoresistance in mouse models, suggesting a potential phage therapy approach for CRC patients with high *B. fragilis* levels
	Viruses: Temperate phages (e.g., Caudoviricetes) Bacteria: Bacterial hosts (e.g., *Enterococcus faecalis*, *Lactobacillus* spp., *Alistipes* spp., *Ruminococcaceae*)	Prophage induction and horizontal gene transfer (HGT): *H. pylori* infection is hypothesized to cause inflammation and oxidative stress, triggering the activation of dormant prophages (temperate phage DNA integrated in bacterial genomes) into a lytic cycle. These phages then transfer auxiliary metabolic genes (AMGs) such as toxin-antitoxin systems, peptidoglycan hydrolases (flgJ), and adhesins (ata, sadA) to bacterial hosts	1. Alters bacterial community composition and physiology 2. Enhances bacterial virulence, survivability, and potentially transforms commensal bacteria into pathobionts 3. Increases the potential for phage-mediated HGT early in carcinogenesis	Drives dysbiosis and potentially supplies bacteria with pro-carcinogenic traits. Phages infecting CRC-promoting bacteria such as *E. faecalis* were highly abundant early in tumorigenesis
	Virus: EBV Bacterium: *H. pylori*	The host's immune response to *H. pylori* infection generates ROS. The bacterial Catalase protein counteracts this by breaking down hydrogen peroxide. The observed higher seroreactivity to Catalase in EBV-positive cancer patients could reflect heightened immune-mediated oxidative stress, which is also a postulated mechanism for reactivating EBV from its latent state	The similar etiological role of *H. pylori* in both EBV-positive and EBV-negative GC, with a borderline specific association of the anti-Catalase response in the EBV-positive subgroup	The similarity in antibody profiles indicates that chronic *H. pylori* infection plays an essential etiological role in gastric carcinogenesis, regardless of the tumor's EBV status. The specific association with Catalase suggests a potential unique interaction where oxidative stress might influence the development of the EBV-positive subtype, but this requires further validation
	Virus: SARS-CoV-2 Bacteria: various gut bacterial populations (e.g., depletion of SCFA-producers, enrichment of pro-inflammatory genera)	The interaction between SARS-CoV-2 and gut bacteria is primarily indirect, mediated through virus-induced host inflammation and ACE2 receptor downregulation, which disrupts gut homeostasis	1. Impairs systemic immune dysregulation (the gut-lung axis) 2. Worsens COVID-19 severity	While not directly tested in this study, the resulting chronic inflammatory state inferentially posing a potential long-term risk for GI carcinogenesis
Archaea-Bacteria	CRC-enriched: • *Methanobrevibacter_A smithii* (Archaea) • *Parvimonas micra* (Bacterium) • *Fusobacterium nucleatum* (Bacterium) CRC-depleted: • *Methanosphaera stadtmanae* (Archaea) • *Methanomassiliicoccus_A intestinalis* (Archaea) • *Roseburia intestinalis* (Bacterium) • *Butyribacter hominis* (Bacterium)	1. Co-occurring/Co-operative interactions: Positive correlations between specific archaea and bacteria 2. Co-excluding/Antagonistic interactions: Negative correlations where the presence of one microbe excludes another 3. Metabolic cross-feeding: Archaea, such as methanogens, consume bacterial fermentation end-products (e.g., hydrogen, acetate). This interspecies metabolite transfer influences the overall metabolic output of the microbial community	1. Altered microbial community structure 2. Shift in metabolic pathways: Enrichment of hydrogenotrophic methanogenesis and depletion of aceticlastic methanogenesis 3. Loss of protective functions: Weakened positive interactions between depleted archaea and beneficial, SCFA-producing bacteria 4. Promotion of pathogenic environment	1. Promotive: The consortium of enriched *M. smithii* and pathogenic bacteria such as *F. nucleatum* could create a pro-tumorigenic environment, potentially promoting CRC development and progression 2. Protective: The loss of archaea such as *M. stadtmanae* and *M. intestinalis*, which normally interact with SCFA-producing bacteria and exclude pathogens, could remove a protective barrier against carcinogenesis, facilitating the growth of harmful bacteria
	CRC-enriched: • *Haloplanus CBA1113* (Archaea) • *Halopelagius longus* (Archaea) • *Bacteroides fragilis* (Bacteria) • *Bacteroides caccae* (Bacteria) • *Oscillibacter* spp. *PEA192* (Bacterium) • *Lachnoclostridium* sp. *YL32* (Bacterium) • *Clostridium bolteae* (Bacterium) • *Alistipes shahii* (Bacterium) CRC-depleted: • *Clostridium beijerinckii* (Bacterium, butyrate-producing) • *Clostridium kluyveri* (Bacterium, butyrate-producing)	1. Co-occurring associations: Positive correlations (mutualism) between CRC-enriched archaea (halophiles) and CRC-enriched bacteria (e.g., *B. fragilis*) 2. Co-exclusive associations: Negative correlations (antagonism) between CRC-enriched archaea and CRC-depleted, protective bacteria (butyrate-producing *Clostridium* spp.) 3. Diversity correlation disruption: A significant positive correlation between archaeal and bacterial alpha diversities observed in healthy controls and adenoma patients was lost in CRC patients, indicating disrupted global cooperation	1. Synergistic network: Co-occurrence between pathogenic archaea and bacteria could create a synergistic, pro-tumorigenic network 2. Loss of protective functions: Co-exclusivity with butyrate-producing bacteria could reduce the production of beneficial SCFAs, which are known to be protective for colonic health 3. Ecological dysbiosis: The disruption of the overall diversity correlation suggests a breakdown in the stable, cooperative ecological structure of the gut microbiome in CRC	1. Promotive: The mutualistic network between CRC-enriched halophilic archaea and oncogenic bacteria (such as enterotoxigenic *B. fragilis*) could synergistically contribute to colorectal carcinogenesis. *B. fragilis*, with its high centrality in the network, could play a key role in this process 2. Promotive: The antagonistic relationship leading to the depletion of butyrate-producing bacteria removes a protective barrier against tumorigenesis. The enrichment of halophiles, potentially driven by a salty diet, could be a mediator in this detrimental shift

*Abbreviations: 1-ABC* 1-Acetyl-β-carboline, *ACE2* angiotensin-converting enzyme 2, *CRC* colorectal cancer, *EBV* Epstein-Barr virus, *GC* gastric cancer, *GI* gastrointestinal, *ROS* reactive oxygen species, *SARS-CoV-2* Severe Acute Respiratory Coronavirus 2, *SCFA* short-chain fatty acid

#### Fungi-bacteria

The role of the mycobiome in carcinogenesis cannot be fully comprehended in isolation. It is progressively examined through the perspective of complex cross-kingdom interactions with the bacteriome. These interactions, encompassing physical adhesion, metabolic exchange, immune modulation, and direct antagonism, collectively influence the microbial environment and demonstrate a fundamental duality in cancer, either enhancing host defense or collaborating in the promotion of tumorigenesis [24, 231–237]. A stable gut microbiota exerts protective effects by actively constraining fungal pathogenicity through multiple mechanisms. A key mode of interaction is metabolic cross-talk, where commensal bacteria such as *Lactobacillus* secrete metabolites, including SCFAs, that directly suppress the yeast-to-hyphal transition of *C. albicans*, a critical virulence determinant [238–240]. This metabolic inhibition is complemented by immune-mediated interactions, whereby SCFAs also bolster anti-fungal T-helper responses, enhancing immune surveillance [239, 240]. Furthermore, bacteria can provide colonization resistance through niche competition. The anaerobe *Bacteroides thetaiotaomicron* competes with fungi and induces epithelial antimicrobial peptides (e.g., LL-37), thereby limiting fungal expansion [214]. The therapeutic potential of this protective symbiosis is demonstrated by the co-administration of *L. kefiranofaciens* and *S. cerevisiae*, which alleviates colitis and carcinogenesis in murine models [241].

In contrast, dysbiosis can promote a network of synergistic, pro-tumorigenic interactions characteristic of CRC. Ecological studies demonstrate specific fungal-bacterial co-abundance networks in CRC, such as *Talaromyces islandicus* with *Clostridium saccharobutylicum* and *Fusobacterium nucleatum* with *Aspergillus rambellii* [64, 242]. These partnerships are supported by intricate inter-kingdom signaling crosstalks. Bacteria such as *Klebsiella pneumoniae* and *Escherichia coli* upregulate *C. albicans* WOR1, thereby priming the white-opaque switch. *Clostridium perfringens* releases heat‑stable signals that induce fungal aggregation via a biofilm transcriptional network [243]. Conversely, fungi can also establish a physical niche for bacterial pathogens. The hypoxic microenvironment within *C. albicans* biofilms facilitates the proliferation of anaerobic pathobionts, such as *Clostridium perfringens* and *B. fragilis* [244]. Additionally, some bacteria could promote their own proliferation through metabolic utilization of fungal components. For instance, *Bacteroides thetaiotaomicron* and other *Bacteroides* species produce specialized enzymes to degrade fungal α-mannans, a major constituent of the fungal cell wall, thereby fueling their own growth and maintaining a dysbiotic microbial community [245, 246]. Such metabolic cross-feeding exemplifies an example of moving beyond co-occurrence to establish a syntrophic interaction. Complementing this metabolic interaction is virulence amplification among known pathogens, such as *F. nucleatum* and *H. pylori*, both of which collaborate with *C. albicans* to boost the consortium’s oncogenic potential [247, 248].

#### Virus-bacteria

The complex interplay between viruses and bacteria constitutes a critical layer of regulation in the cancer microbiome, influencing microbial ecology, host immunity, and ultimately, tumour progression. Bacteriophages exert a profound influence on bacterial ecology through their dynamic life cycles, which include lysogenic and lytic phases [249]. In maintaining homeostasis, temperate phages integrate as prophages, conferring advantages such as antibiotic resistance and metabolic fitness to their bacterial hosts, thereby enhancing their ecological competitiveness [250]. This symbiotic relationship is stringently regulated. For instance, environmental stressors such as antibiotics or pH fluctuations can induce prophages into the lytic cycle, a process that functions as a key mechanism for controlling bacterial population densities and sustaining community stability [251–253]. However, in a dysbiotic state, extensive phage-mediated lysis occurs, depleting specific bacterial populations by 40%–60% and potentially aggravating inflammation and microbial imbalance [254]. The therapeutic potential of this targeted lysis is exemplified by phage vB_BfrS_VA7 (VA7) [255, 256], which selectively reduces the burden of oncogenic *B. fragilis* in murine models of CRC without adverse effects [257]. Confirming correlation-based network analyses, this study experimentally validates that *B. fragilis* directly interacts with CRC cells through surface its SusD/RagB binding to Notch1 expressed on CRC cells, an inter-kingdom signaling pathway that triggers chemoresistance, whilst phage VA7 selectively eradicates this pathobiont, thereby functionally establishing causality rather than merely providing an association derived from network analysis [257]. The clinical significance of phage-bacteria networks in cancer is further emphasized by the enrichment of specific phages targeting *F. nucleatum* and other bacteria associated with CRC in patients [258], as well as by the application of phage display technology to develop *F. nucleatum*-targeting probes (e.g., M13 phage) [259].

Direct synergistic partnerships between eukaryotic viruses and bacteria can markedly accelerate carcinogenesis. The interaction between EBV and *H. pylori* in GC exemplifies a paradigm, although the nature of their relationship is intricate. While some epidemiological investigations suggest an inverse correlation, potentially indicating competitive exclusion [260, 261], functional analyses demonstrate a significant oncogenic synergy. Co-infection correlates with severe inflammation and an increased risk of intestinal-type GC and precancerous lesions [262]. Mechanistically, the interaction between EBV and *H. pylori* amplifies the oncogenic characteristics of gastric cells, promoting increased focus formation, cellular migration, and proliferation, a process partly mediated by the upregulation of the oncogenic protein gankyrin [263]. Viral infections can also promote a pro-tumorigenic state by indirectly reshaping the gut microbiota. SARS-CoV-2 infection presents a prominent example, causing notable alterations in the gut microbiota. These changes are characterized by a depletion of the genera *Ruminococcus*, *Alistipes*, *Eubacterium*, *Bifidobacterium*, *Faecalibacterium*, *Roseburia*, *Fusicathenibacter*, and *Blautia*, alongside an enrichment of *Eggerthella*, *Bacteroides*, *Actinomyces*, *Clostridium*, *Streptococcus*, *Rothia*, and *Collinsella* [264]. Critically, this SARS-CoV-2-associated dysbiosis is not merely correlational. It drives increased colonic inflammation, compromises the gut barrier, and upregulates gene expression pathways involved in CRC tumorigenesis and immunosuppression, thereby creating a microenvironment that exacerbates cancer progression [265].

#### Archaea-bacteria

Archaea, historically neglected components of the gut microbiome, are now recognized as pivotal regulators of microbial ecology in CRC. Their impact is realized not in isolation but via a complex network of interactions with bacteria, establishing a metabolic and ecological environment conducive to tumour development. Large-scale multicohort metagenomic analyses have begun to map this intricate network, positioning specific archaea as central players. *B. fragilis* demonstrates the highest number of positive correlations with CRC-enriched archaea and exhibits elevated node betweenness centrality, suggesting its role as a pivotal hub within this dysbiotic ecosystem [87]. Conversely, CRC-depleted methanogenic archaea demonstrated concurrent interactions with SCFA-producing bacteria, such as *Roseburia intestinalis*, *Butyribacter hominis*, and *Agathobacter rectalis*, while exhibiting mutually exclusive correlations with potential CRC pathogenic bacteria, including *F. nucleatum*. The intensification of these correlation patterns from HCs to adenoma and CRC sequences implies a dynamic restructuring of the archaeal-bacterial interactome that parallels disease progression [88].

The fundamental underpinning of this interaction is a metabolic symbiosis centered on hydrogen and metabolite exchange. Methanogenic archaea consume bacterial fermentation end-products, thereby preventing metabolite accumulation and promoting microbial metabolism toward optimal SCFA synthesis, a process vital for colonic homeostasis [57, 266, 267]. This syntrophic relationship is further enhanced through direct interspecies interactions, wherein bacteria supply methanogens with electron donors, and archaea, in turn, provide a stable metabolic sink, facilitated by electron shuttles that improve the efficiency of this cooperation [268]. Similar archaea–bacterial metabolic interdependencies, driven by environmental stress and genome size disparity, have been observed in thermophilic communities, where heat promotes commensalistic cross-feeding [269]. At the molecular level, archaea employ sophisticated mechanisms to interact with their environment and other microbes. Biofilm-forming archaea produce extracellular polymeric substances (EPS), which serve as scaffolds for cell adhesion and facilitate the formation of multi-kingdom consortia [270]. The production of EPS, as observed in organisms such as *Halobacterium salinarum*, also offers protection against environmental stressors, potentially improving survival within the TME [271]. Furthermore, communication mechanisms such as quorum sensing (QS) allow archaea to detect population density and regulate their behavior in a coordinated manner [272], although the specific signaling molecules and pathways in the gut context continue to be a promising area for future investigation [273].

## Translational and clinical implications

**Fig. 4:**
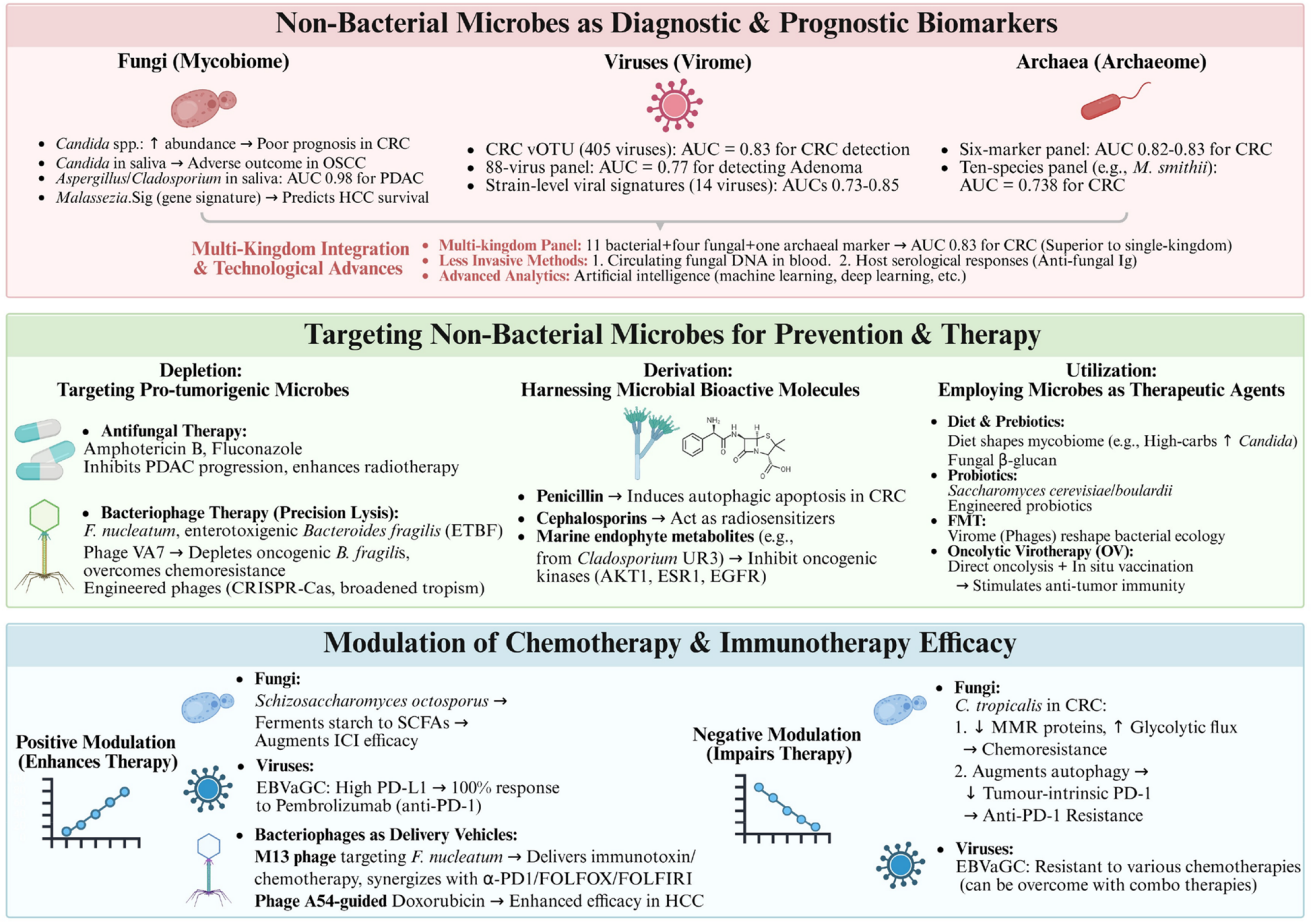
Translational applications of non-bacterial microbes: biomarkers, therapeutic targets, and modulators of treatment response. AUC, area under the curve; CRC, colorectal cancer; HCC, hepatocellular carcinoma; OSCC, oral squamous cell carcinoma; PDAC, Pancreatic Ductal Adenocarcinoma; vOTU, viral Operational Taxonomic Unit

The investigation of non-bacterial microbes is swiftly progressing from a matter of biological curiosity to practical clinical application. These organisms possess substantial potential as innovative diagnostic biomarkers, prognostic indicators, and therapeutic targets. Additionally, they play a crucial role in influencing the effectiveness of traditional chemotherapy and immunotherapy, thereby opening new pathways for precision oncology and combined treatment strategies (Fig. 4 and Table 3).

**Table 3:** Harnessing the non-bacterial microbiome: From bench to bedside

Application area	Targets	Strategy	Mechanism	Associated cancers	Current status	Challenges
Biomarkers	*Candida*-to-*Saccharomyces* (C/S) ratio	Calculate the ratio of Candida to *Saccharomyces* DNA abundance from tumor tissue metagenomic data	A high C/S ratio is associated with late-stage (Stage IV) and metastatic disease, potentially linked to loss of epithelial barrier function	Colon cancer	Research discovery (identified as a significant association within the TCGA cohort)	Requires defining standardized and clinically actionable cut-off values
	*Candida* spp.	Detection of circulating fungal DNA in blood plasma	Fungal DNA could translocate from GI tumors into the bloodstream, potentially due to deteriorated epithelial barrier function. The mycobiota composition in blood was significantly similar to patient-matched tumors	GI cancer	Preliminary research finding (correlative observation within the TCGA cohort; not experimentally validated in blood)	1. Very low abundance in blood 2. High risk of contamination during sampling 3. Requires extensive validation to confirm tumor origin vs. other sources
	11 bacterial (e.g., *Fusobacterium nucleatum*, *Parvimonas micra*), four fungal (e.g., *Talaromyces islandicus*, *Aspergillus rambellii*), and one archaeal species	Multi-kingdom metagenomic analysis and machine learning	Bacterial-fungal interactions, upregulated D-amino acid and butanoate metabolism	CRC	Validated across eight cohorts, AUROC 0.83 for CRC, 0.78 for early-stage	1. Geographical heterogeneity 2. Clinical translation
	Fungi: *Schizosaccharomyces octosporus*, *Trichophyton benhamiae*, others (26-taxa core)	Using machine learning on gut metagenomic data to create predictive models of ICI response based on single- and multi-kingdom microbial biomarkers	1. Modulation of tumor immune microenvironment (enriched exhausted CD8^+^ T cells) 2. Proposed microbial metabolite production (e.g., SCFAs from starch fermentation) 3. Bacterial-fungal inter-kingdom interactions	Pan-cancer: Melanoma, non-small cell lung cancer, renal cell carcinoma, GI cancers	Translational research phase Retrospective analysis of multiple cohorts with biological validation in an independent cohort	1. Technical variability between studies 2. Establishing causality from correlation 3. Translation into clinical practice
	A combination of four viral genera: *Betabaculovirus*, *Epsilon15likevirus*, *Mulikevirus*, *Punalikevirus*	Quantification of the abundance of specific viral taxa from fecal metagenomic data to calculate a risk score	The abundance of these specific viral taxa is associated with patient survival outcomes. The biological mechanism is not elucidated but could be related to phage-bacteria interactions influencing the TME or disease progression	CRC	Research discovery	1. The biological driver vs. passenger role of these viruses is not established 2. Requires validation in larger, prospective cohorts
	405 CRC-associated vOTUs	Meta-analysis of nine cohorts (1,282 samples)	CRC-enriched vOTUs encode enzymes such as N-acetylmuramoyl-L-alanine amidase (disrupts biofilms)	CRC	Prediction models show high accuracy (average AUC: 0.830)	1. Low occurrence rate of CRC-enriched vOTUs in fecal samples 2. Mechanisms of viral impact on carcinogenesis are not fully understood 3. Requires experimental validation (in vitro/in vivo)
	- Enriched in CRC: *Methanobrevibacter_A smithii*, *Methanobrevibacter_A sp002496065* - Depleted in CRC: *Methanosphaera stadtmanae*, *Methanomassiliicoccus_A intestinalis*, and six other archaeal species	1. Multi-cohort metagenomic analysis of 2,101 samples from 11 cohorts across seven countries 2. Taxonomic profiling using a customized Kraken2 database built on the Genome Taxonomy Database (GTDB) 3. Machine learning with a Random Forest model and leave-one-cohort-out validation to identify and test a multi-kingdom diagnostic biomarker panel	1. Functional shift: Enrichment of methanogenesis-related genes and pathways (e.g., hydrogenotrophic methanogenesis) in CRC 2. Ecological interactions: Altered co-occurring and co-excluding interactions between archaea and bacteria. CRC-depleted archaea showed enhanced positive correlations with butyrate-producing bacteria and negative correlations with CRC-enriched pathogenic bacteria	CRC	1. Research phase. The combined archaeal and bacterial biomarker model showed robust diagnostic potential in a multi-cohort validation 2. Performance: The combined model achieved an AUC of up to 0.931 in leave-one-cohort-out analysis, outperforming models using bacteria alone	1. Inter-cohort variation: The archaea-based biomarker model showed varying performance across different geographic cohorts 2. Mechanistic understanding: The study is an association analysis; further experimental investigations are needed to establish a causal relationship and unravel the precise mechanistic role of archaea in colorectal tumorigenesis
Novel therapeutics	Commensal fungi (e.g., Saccharomycetales order, including *Candida albicans* and *Saccharomyces*)	Depletion of intestinal fungi using antifungal antibiotics (e.g., fluconazole, 5-fluorocytosine)	The depletion of fungi reduces immunosuppressive signals via the Dectin-1 receptor on tumor-associated macrophages, leading to decreased PD-1^+^ T cells, reduced pro-tumor macrophages (CD206^+^F4/80^+^), and enhanced CD8^+^ T cell cytotoxicity and tumor cell death post-radiation therapy	Breast cancer, Melanoma	Preclinical (mouse models)	1. Understanding complex fungal-bacterial interactions 2. Translating antifungal adjunct therapy to human trials 3. Potential for antifungal resistance 4. Identifying specific pathogenic fungal species vs. overall community balance
	Commensal fungi, specifically *Alternaria alternata* and *Malassezia globosa*	1. Genetic deletion of IL-33 in cancer cells using shRNA or CRISPR-Cas9 2. Depletion of the mycobiome using oral antifungal therapy (amphotericin B or fluconazole)	The intratumoral mycobiome (fungi/fungal components) activates the Dectin-1-Src-Syk-CARD9 pathway in cancer cells, triggering the secretion of IL-33 from the nucleus into the extracellular space. Secreted IL-33 recruits and activates TH2 cells and innate lymphoid cells 2 (ILC2s) in the TME. These cells secrete pro-tumorigenic cytokines (IL-4, IL-5, IL-13), promoting tumor progression	PDAC	Preclinical research conducted in genetically engineered mouse models and human tissue samples	1. Understanding the precise molecular link between fungal components and IL-33 secretion 2. Determining the point in tumorigenesis when fungal retrograde transfer from the gut to the pancreas occurs 3. The potential presence of fungal components (not live fungi) could render prolonged antifungal therapy ineffective
	Commensal fungi, specifically *Malassezia* spp. (e.g., *M. globosa*)	Ablation of the mycobiome using oral antifungal agents (amphotericin B or fluconazole)	Fungi migrate from the gut to the pancreas. *Malassezia* spp. activate the MBL pathway, which triggers the complement cascade (via C3 convertase leading to C3a production). C3a binds to its receptor (C3aR) on PDAC cells, promoting tumor cell proliferation and progression	PDAC	Preclinical research conducted in genetically engineered mouse models and human tissue samples	1. Establishing whether fungal dysbiosis is a cause or consequence of oncogenesis 2. Understanding the dynamic crosstalk between the mycobiome and the bacterial microbiome
Microbiome modulation	Fungal species: *Candida tropicalis* and *Candida albicans*	Using a novel, designed probiotic formulation delivered as a cell-free filtrate	Inhibition of biofilm formation and disruption of mature biofilms Inhibition of fungal virulence	CRC	The research has progressed through in vitro experiments demonstrating efficacy in preventing and treating pathogenic polymicrobial biofilms	1. Identification of active components 2. Clinical validation
	Fungal species: *Saccharomyces boulardii*	using *Saccharomyces boulardii* (or its culture supernatant) to inhibit pathological angiogenesis	Inhibition of VEGFR signaling, specifically through reduced phosphorylation of VEGFR-2 and suppression of downstream kinases PLCγ and ERK1/2, leads to inhibition of angiogenesis (new blood vessel formation)	IBD and highly relevant to cancer therapy	Findings are based on cell culture and animal models	Understanding the diverse and complex interactions of probiotics with the intestine and its microbiota
	Alleviates microbiota dysbiosis, increases beneficial bacteria	Oral administration of *Ganoderma lucidum* polysaccharide (GLP) as a prebiotic	1. Modulates gut microbiota: Increases abundance of SCFA-producing bacteria 2. Increases SCFAs: Elevates levels of butyric, propionic, and isobutyric acids in colon and serum 3. Immunomodulation: - Activates antitumor immunity: Increases cytotoxic CD8^+^ T cells and Th1 cells; decreases immunosuppressive Tregs - Downregulates IDO enzyme activity and serum Kyn/Trp ratio 4. Synergizes with anti-PD-1: Enhances checkpoint inhibitor's effect by further improving T-cell function and reducing Treg infiltration	CRC	Preclinical research Efficacy demonstrated in vivo	1. Model limitations 2. Mechanistic depth: Exact molecular pathways linking microbial changes (e.g., butyrate) to immune cell activation require further elucidation 3. Translation to humans 4. GLP characterization: The polysaccharide's structure and composition can vary based on extraction methods, potentially impacting activity and consistency
	Prebiotics	Using an oral liquid formulation composed of polysaccharides from *Lentinus edodes*, *Ganoderma lucidum*, and *Poria cocos* to inhibit tumor growth	Downregulation of the anti-apoptotic protein Bcl-2 and upregulation of the pro-apoptotic proteins caspase-9 and caspase-3, thereby inducing apoptosis in sarcoma cells	Sarcoma	Preclinical research	Mechanisms of polysaccharides are still unclear completely
	Dietary supplementation	Oral administration of a defined cocktail of four bacteriophages (PreforPro®: LH01-*Myoviridae*, LL5-*Siphoviridae*, T4D-*Myoviridae*, LL12-*Myoviridae*) as a daily dietary supplement	1. Direct lysis: Bacteriophages specifically infect and lyse their target host bacteria (*E. coli*), reducing its population 2. Ecological modulation: By reducing specific bacteria, the treatment could alter the gut ecosystem, potentially creating space for beneficial bacteria to thrive (e.g., butyrate producers) 3. Immunomodulation: The reduction in target bacteria could lead to decreased bacterial pro-inflammatory components (e.g., LPS), resulting in lower systemic inflammation (e.g., reduced IL-4)	CRC	Proof-of-concept in healthy adults	1. Variable baseline microbiota 2. Inter-individual variability 3. Lack of dietary control
	Dietary supplementation	Oral administration of a synergistic combination: A daily dose of a probiotic (1 × 10^9^ colony forming units of *B. lactis* BL04) together with a cocktail of *E. coli*-targeting bacteriophages (PreforPro: 1 × 10^6^ plaque forming units of LH01-*Myoviridae*, LL5-*Siphoviridae*, T4D-*Myoviridae*, LL12-*Myoviridae*)	1. Direct lysis: The phages lyse their primary target (*E. coli*), potentially reducing competition for resources and space 2. Enhanced probiotic activity: This modified gut environment could potentiate the activity and growth of the co-administered probiotic (*B. lactis*) and other beneficial commensals (e.g., *Lactobacillus*), which showed a tenfold increase with the combination therapy 3. Reduction of pro-inflammatory taxa: The treatment led to a decrease in bacteria associated with gut inflammation (*Citrobacter*, *Desulfovibrio*), thereby improving the intestinal environment	CRC	Phase II human clinical trial	1. Low and variable phage detection 2. Complexity of mechanism 3. Power and measurement: The study could have been underpowered to detect all significant differences in GI symptoms between groups, and stool consistency measurements had limitations
Adjunct to existing therapy	Commensal fungi, specifically *Malassezia* spp. (e.g., *M. globosa*)	Ablation of the mycobiome using oral antifungal agents (amphotericin B or fluconazole) prior to and in combination with chemotherapy	Depleting the oncogenic mycobiome removes its immunosuppressive and tumor-promoting effects (mediated via the MBL-C3 complement cascade). This is hypothesized to reshape the TME, potentially making it more permissive to the cytotoxic effects of chemotherapy	PDAC	Preclinical evidence from mouse models	The exact immune or cellular mechanisms by which mycobiome ablation synergizes with chemotherapy are not fully elucidated
	Fungal mycobiome (*Candida tropicalis*)	Targeting the interaction between *C. tropicalis* and tumor cell autophagy to prevent PD-1 downregulation	1. Autophagy induction 2. PD-1 downregulation 3. Promoted tumor growth 4. Therapy resistance: This mechanism could explain partial lack of response to anti-PD-1 checkpoint blockade in some patients, as the treatment further blocks a tumor-suppressive signal	CRC	Preclinical research	1. Translating to humans 2. Microbiome complexity 3. Therapeutic targeting 4. Treatment stratification 5. Combination therapies
	Fungal mycobiome (*Candida tropicalis*)	Inhibiting lactate production or signaling to restore MMR function and chemosensitivity	1. Enhanced glycolysis 2. Lactate signaling 3. Inhibition of MMR 4. Chemoresistance: Downregulation of MLH1 (and MSH2) impairs the MMR system, allowing cancer cells to accumulate mutations and survive oxaliplatin-induced DNA damage, leading to treatment resistance	CRC	Preclinical research	1. Translational gap 2. Therapeutic targeting 3. Microbiome complexity 4. Patient stratification 5. Treatment combination
	*Bacteroides fragilis* (both enterotoxigenic and non-toxigenic strains)	Precision phage therapy using the strictly lytic phage vB_BfrS_VA7 (VA7) to selectively eliminate *B. fragilis* in the gut microbiome	1. Chemoresistance mechanism: *B. fragilis* colonizes CRC tumors. Its surface protein SusD/RagB binds directly to the Notch1 receptor on CRC cells, activating the Notch1 signaling pathway. This leads to epithelial-to-mesenchymal transition (EMT), increased cancer stemness, and suppression of chemotherapy-induced apoptosis 2. Therapeutic intervention: Phage VA7 specifically infects and lyses *B. fragilis*, thereby disrupting the SusD/RagB-Notch1 interaction and restoring sensitivity to chemotherapy-induced cell death	CRC	Preclinical validation. The study provides robust evidence from: 1. Human cohorts 2. In Vitro Models 3. In Vivo Models 4. Safety	1. Translation to humans: Efficacy and safety of phage VA7 need to be confirmed in clinical trials with CRC patients 2. Complex microbiome: The broader ecological impact of selectively eliminating a single species in the human gut microbiome requires further investigation 3. Manufacturing and regulation: Developing phage therapies as standardized, approved drugs presents regulatory and production hurdles
	Phage A54 (sequence AGKGTPSLETTP)-derived peptide	1. Peptide-drug conjugation: The identified specific peptide (A54) was chemically synthesized and conjugated to the chemotherapeutic drug DOX to create a targeted therapeutic (A54-DOX) 2. Validation: The binding specificity of the peptide and the efficacy of the conjugate were tested in vitro and in vivo	1. Targeting: The A54 peptide (sequence: AGKGTPSLETTP) specifically binds to a receptor(s) on the surface of HCC cells 2. Drug delivery: When conjugated to DOX, the A54 peptide acts as a homing device, directing the cytotoxic drug specifically to the tumor cells 3. Therapeutic effect: This targeted delivery increases the local concentration of DOX within the tumor, leading to enhanced cancer cell death and tumor growth inhibition, while potentially reducing systemic side effects	HCC	Preclinical Research	1. Unknown receptor 2. Residual non-specific toxicity 3. Translation to humans 4. Optimization required: The authors suggest that coupling the A54 peptide to a toxin engineered to lack non-specific cell binding (e.g., a truncated Pseudomonas exotoxin) could further improve the therapeutic window
	Delivery vehicle: Non-pathogenic strains of *Escherichia coli* (*E. coli* ER2738 and engineered *E. coli* Nissle 1917 (EcN)) Therapeutic agent: Engineered M13 filamentous bacteriophage	A combination therapy using two engineered bacterial strains for targeted, intratumoral delivery of therapeutic agents 1. E-Phage strain: *E. coli* engineered to produce and release an M13 bacteriophage displaying the PD-1 protein on its surface 2. E-CPE strain: *E. coli* Nissle 1917 engineered with a lysis system to produce and controllably release an immunotoxin (CPE) upon induction	1. Tumor colonization: Both engineered strains naturally target and colonize tumor tissues after intravenous injection 2. Localized drug release: - E-Phage releases PD-1-displaying phages that bind to PD-L1 on tumor cells, blocking the PD-1/PD-L1 immune checkpoint and reactivating T-cells - E-CPE is induced by L-arabinose to lyse and release the CPE immunotoxin, which binds to CD47 on tumor cells and induces direct cell death 3. Synergistic effect: The combination reactivates the immune system (via PD-1-phage) and directly kills tumor cells (via CPE), leading to enhanced antitumor immunity and tumor suppression	CRC	Preclinical Research	1. Safety 2. Drug resistance 3. Model limitations 4. Immune response complexity: The study primarily focused on CD4^+^ T-cells; a more comprehensive analysis including CD8^+^ T-cells is needed 5. Clinical translation
	EBV	Combination therapy (Sequential application of the PI3K inhibitor LY294002 following 5-FU treatment)	1. 5-FU induces resistance: 5-FU monotherapy upregulates phosphorylated AKT (p-AKT) and phosphorylated NF-κB (p-NFκB) in EBV-positive gastric cancer cells, promoting cell survival and chemoresistance 2. LY294002 inhibits survival pathway 3. Synergistic effect: Sequential treatment (5-FU followed by LY294002) synergistically induces cytotoxicity	EBV-associated gastric cancer	Preclinical Research	1. The research is at an early stage, confined to cell line models 2. The treatment schedule is critical; the study found the synergistic effect was specific to the sequence of 5-FU followed by LY294002 3. Potential toxicity and efficacy of this combination regimen in humans are unknown

*Abbreviations*: *5-FU* 5-fluorouracil, *AUC* area under the curve, *AUROC* area under the receiver operating characteristic curve, *CRC* colorectal cancer, *DOX* Doxorubicin, *EBV* Epstein–Barr virus, *GI* gastrointestinal, *HCC* hepatocellular carcinoma, *IBD* inflammatory bowel disease, *ICI* immune checkpoint inhibitor, *MBL* mannose-binding lectin, *MMR* mismatch repair, *PDAC* pancreatic ductal adenocarcinoma, *PD-1* programmed cell death 1, *SCFA* short-chain fatty acid, *TCGA* The Cancer Genome Atlas, *TME* tumor microenvironment, *VEGFR* vascular endothelial growth factor receptor, *vOTUs* viral operational taxonomic units

### Non-bacterial microbes as diagnostic and prognostic biomarkers

The non-bacterial microbiome has emerged as a rich and largely untapped reservoir of diagnostic and prognostic biomarkers for GI cancers. Moving beyond bacteria, signatures derived from fungi, archaea, and viruses demonstrate significant clinical potential, both independently and in integrated models. Individually, each microbial kingdom harbors robust biomarkers. In CRC, a high abundance of *Candida* species is strongly correlated with advanced, metastatic disease and serves as an independent predictor of poor survival [27]. The diagnostic power of fungal and archaeal signatures is further evidenced by their ability to distinguish CRC patients from healthy individuals, with mycobiome-based (14 markers) and archaea-based (six markers) panels achieving area under the curve (AUC) values of 0.74–0.93 [63] and 0.82–0.83 [87], respectively. Crucially, a large-scale, multi-cohort study by Li et al. provided robust statistical validation for the clinical utility of gut archaea as non-invasive diagnostic biomarkers for CRC [88]. By analyzing 2,101 metagenomes, the authors identified a universal panel of 10 archaeal species that distinguished CRC patients from HCs with an AUC of 0.738 in pooled data, with *Methanobrevibacter_A smithii*, *Methanomassiliicoccus_A intestinalis*, and *Methanosphaera stadtmanae* emerging as the top contributing features. Most notably, the study demonstrated the immense power of a multi-kingdom approach. A combined model incorporating the 10 archaeal biomarkers alongside 35 bacterial biomarkers achieved a significantly superior diagnostic performance compared to models based on bacteria alone. In rigorous leave-one-cohort-out (LOCO) cross-validation analyses across 11 independent cohorts, this combined archaeal-bacterial panel yielded AUCs ranging from 0.744 to 0.931, consistently outperforming single-kingdom models [88]. This cross-continental validation firmly establishes gut archaea, particularly when integrated with bacterial signatures, as powerful and generalizable biomarkers for non-invasive CRC detection. This prognostic value extends to other GI malignancies. In OSCC, high salivary *Candida* levels portend adverse outcomes, whereas *Malassezia* is associated with a more favorable prognosis [129]. Similarly, the salivary mycobiome exhibits remarkable classification capability for PDAC, with *Aspergillus* and *Cladosporium* achieving exceptional AUCs of 0.983 and 0.969, respectively [274]. In a 2025 study, investigators translated this ecological observation into a host-response biomarker, developing a *Malassezia*-derived gene signature (*Malassezia*.Sig) that accurately predicts HCC patient survival [92]. This approach aligns with other successful prognostic models based on microbial-influenced metabolic genes in HCC and gastric cancer [275, 276]. While these individual kingdoms provide substantial diagnostic and prognostic value, the integration of multiple kingdoms signifies a substantial advancement. A comprehensive multi-kingdom stool panel, incorporating 11 bacterial, four fungal, and one archaeal marker, attained an average AUC of 0.83 for CRC detection. This performance exceeds that of any individual kingdom panel under comparable conditions [242], thereby highlighting the superior diagnostic predictive capacity of an ecosystem-wide perspective.

Concurrently, less invasive approaches are gaining traction. The detection of circulating fungal DNA in blood allows for modest differentiation of cancer types and stages [65], while profiling host serological responses (e.g., immunoglobulins targeting specific fungi) offers a promising indirect method for cancer stratification [277, 278]. Advances in shotgun metagenomics and machine learning are powerfully extending these principles to the virome [279]. Taxa-level (22 taxa, AUCs 0.72–0.80) or strain-level viral gene expression signatures (14 viruses, AUCs 0.73–0.85) are capable of discriminating between patients with CRC and individuals without the disease in independent cohorts [70]. Large-scale meta-analyses have consolidated these findings, defining a CRC-associated vOTU of 405 viruses (AUC = 0.83) and an 88-virus panel capable of detecting precancerous adenomas (AUC = 0.77) [280]. Notably, the recent advent of artificial intelligence (AI) foundation models and “copilots” in cancer pathology has showcased remarkable potential for improving diagnostic accuracy and integrating complex data modalities [281]. Extending such methodologies to incorporate multi-kingdom microbial biomarkers represents a promising but yet-to-be-realized direction for future research.

### Targeting non-bacterial microbes for prevention and therapy

The burgeoning understanding of non-bacterial microbes in the field of oncology has prompted the development of new therapeutic strategies, which range from direct microbial eradication to their innovative use as therapeutic agents. These strategies can generally be classified into three paradigms: depletion, derivation, and utilization (Section “Harnessing diet, probiotics/prebiotics, FMT, bacteriophages, and oncolytic virotherapy for microbial interventions”).

Direct targeting of pro-tumorigenic fungi and viruses constitutes a straightforward therapeutic approach. In PDAC, antifungal agents such as amphotericin B and fluconazole have demonstrated significant inhibition of tumour progression in murine models [28, 29]. This strategy is especially compelling given the intricate interactions among bacterial and fungal communities. As Shiao et al. [30] elucidated, depletion of fungi enhances responsiveness to radiotherapy. Conversely, broad-spectrum antibiotic treatment results in fungal overgrowth and reduces therapeutic efficacy, thereby emphasizing the importance of meticulous microbial management in combination therapies.

The fungal kingdom represents a prolific source of bioactive molecules with serendipitous anti-cancer properties. Classic beta-lactam antibiotics exemplify this phenomenon. Penicillin, produced by *Penicillium chrysogenum/rubens*, beyond its antimicrobial role, has been shown to interfere with mitochondrial function in colon cancer cells, thereby inducing autophagic apoptosis and reducing metastasis [282]. Cephalosporins, derived from a metabolite of the mold *Cephalosporium coronarium,* have demonstrated potential as radiosensitizers, potentially by exacerbating oxidative damage in cancerous cells [283]. Moreover, the exploration of fungal biodiversity, particularly from unique ecological niches such as marine endophytes, is revealing a wealth of anti-cancer metabolites. The *Cladosporium* spp. UR3 strain, isolated from a sponge in the Red Sea, produces metabolites that effectively inhibit key oncogenic kinases (AKT1, ESR1, EGFR) and exhibits significant activity against CRC [284–286], underscoring the untapped potential of fungal natural products in pharmacological research.

### Harnessing diet, probiotics/prebiotics, FMT, bacteriophages, and oncolytic virotherapy for microbial interventions

#### Diet

The diet functions as a primary environmental determinant of the gut mycobiome, providing a non-invasive method to influence fungal community structure and function for the purposes of cancer prevention and treatment [287]. The composition of dietary macronutrients significantly impacts fungal ecology. For instance, the abundance of *Candida* exhibits a positive correlation with high-carbohydrate diets, whereas it is negatively associated with diets rich in protein, amino acids, and fatty acids [288, 289]. This dietary influence also extends to reducing cancer risk, as the presence of aflatoxin-producing *Aspergillus* spp., a recognized risk factor for HCC, shows a negative correlation with the consumption of SCFAs [289, 290]. Beyond shaping microbial populations, specific fungal components derived from the diet demonstrate direct anticancer properties. A prominent example is fungal β-glucan, a pattern recognition molecule that binds to receptors on innate immune cells (e.g., dectin-1) to initiate phagocytosis, stimulate ROS production, and induce inflammatory cytokines [291–293]. More significantly, particulate yeast-derived β-glucan can reprogram the immunosuppressive TME by promoting the polarization of M2-like tumour-associated macrophages towards an immunostimulatory M1-like phenotype [294]. This macrophage reprogramming, combined with the activation of NK cells, underpins its efficacy in inhibiting colon cancer metastasis [295]. The inherent antioxidative properties of *S. cerevisiae*-derived β-glucan further enhance its protective role against cellular damage [296, 297], thereby positioning it as a promising dietary supplement for adjunctive cancer therapy.

#### Probiotics

The therapeutic application of live microorganisms, or probiotics, represents a promising strategy to reconfigure the TME and inhibit cancer progression. This approach has evolved from leveraging traditional yeast probiotics to the frontier of genetically engineered bacterial agents, offering a versatile platform for cancer management.

Yeasts of the *Saccharomyces* genus are among the most thoroughly characterized probiotics, demonstrating efficacy beyond the management of GI disorders such as diarrhea and inflammatory bowel disease [298, 299]. In CRC, *S. cerevisiae* exerts direct anti-tumour effects by promoting apoptosis in cancer cells and mitigating disease progression through modulation of the mucosal microbiota [300]. Remarkably, even non-viable forms maintain substantial bioactivity. Heat-killed *S. cerevisiae* induces apoptosis and exhibits anti-proliferative effects in CRC cells via downregulation of RelA and upregulation of PTEN [301]. Its therapeutic potential is further amplified when used in conjunction with combination therapies. Specifically, when administered alongside curcumin-loaded niosomal nanoparticles, it synergistically inhibits CRC progression by downregulating metastasis-associated genes (MMP2, MMP9, COL10A1) and promoting cell cycle arrest [302]. Similarly, *Saccharomyces boulardii* has demonstrated direct anti-tumour properties in human colonic cancer cells, whereby it inhibits EGF-induced proliferation, reduces colony formation, and promotes apoptosis [303]. In addition to these direct effects, *S. boulardii* modulates the TME by regulating angiogenesis through VEGFR signaling, thereby alleviating intestinal inflammation and supporting mucosal repair [304]. Moreover, innovative probiotic formulations are under development to target pathogenic microbial communities. Certain strains possess the capability to inhibit the formation of, and even treat, existing *Candida*-bacterial biofilms, offering a novel strategy for managing biofilm-associated GI pathologies, including CRC [305].

The field is undergoing a transformation due to the emergence of genetically engineered probiotic microbiota. These engineered microbes serve as advanced in situ therapeutic agents, capable of producing elevated levels of bioactive molecules directly within the TME. A significant application of this technology involves designing probiotics to secrete immunostimulatory agents, thereby acting as delivery vehicles that markedly improve the infiltration and activation of tumour-infiltrating T cells. This approach has shown notable synergistic effects when used in conjunction with PD-L1 blocking antibodies, resulting in substantial tumour elimination in preclinical models and signaling a new chapter in combination immuno-microbial therapy [306, 307].

#### Prebiotics

Prebiotics, defined as non-digestible compounds that offer health benefits through their fermentation by gut microbiota, constitute a significant approach for the modulation of microbial communities [308]. Fungi, notably medicinal mushrooms, serve as a rich and sustainable source of novel prebiotics with established anti-cancer properties [309–313]. Mushrooms such as *Lentinula edodes *[314], *Trametes versicolor* [315], *Grifola frondose* [316]*, Hericium erinaceus* [317], *Fomes fomentarius* [318] are reported to exhibit potent anti-cancer efficacy. An exemplary compound is *Ganoderma lucidum* polysaccharide (GLP), which inhibits the progression of CRC via a complex mechanism: it boosts anti-tumour CD8^+^ and Th1 immune responses, suppresses Tregs, rectifies microbial dysbiosis by elevating SCFAs, and collaborates with anti-PD-1 immunotherapy [319–322]. The translational advancement of such compounds is exemplified by the Chinese Food and Drug Administration (SFDA)-approved drug “*Poria cocos* polysaccharides oral liquid”, which induces apoptosis in tumour cells by modulating levels of Bcl-2, caspase-3, and caspase-9 proteins, and is utilized either as monotherapy or in combination with standard treatments for cancer [323–325].

Apart from traditional prebiotics, the virome, specifically bacteriophages, is emerging as a pivotal target for maintaining microbial equilibrium. Phages offer a paradigm shift from broad-spectrum modulation to precision editing of the gut microbiota. By selectively lysing pathogenic bacteria, they reduce harmful bacterial loads while creating a niche for beneficial microbes to flourish, effectively acting as targeted ecological engineers [249, 326]. Clinical evidence supports this potential. A 2019 trial (NCT03269617) demonstrated that the PreforPro® *E. coli* phage cocktail significantly reduced fecal *E. coli* without disrupting overall microbial diversity. Importantly, it increased butyrate-producing bacteria, reduced *C. perfringens*, and lowered inflammatory markers, indicating a restorative shift in the gut environment [327]. Furthering this concept, a phase II trial (NCT04511221) showed that a four-week regimen of PreforPro® combined with *Bifidobacterium bifidum* BL04 significantly augmented the abundances of both *Lactobacillus* and the co-administered probiotic, suggesting that phages can act as probiotic synergists, or “phage-biotics” [328]. This synergy is now being leveraged commercially in products such as InnovixLabs® [329] and BioSchwartz® Probiotics [330], which integrate PreforPro® to enhance probiotic efficacy. Through selective bacterial lysis and the subsequent release of cellular components, such phage-probiotic combinations establish an integrated, precision microbiota-modulating system, representing a next-generation solution for gut health and cancer therapy.

#### Faecal microbiota transplantation (FMT)

Faecal microbiota transplantation (FMT), the infusion of processed fecal material from a healthy donor into a patient’s GI tract, constitutes the most direct method for re-establishing a functional gut microbiome [331]. Although its effectiveness in treating recurrent *Clostridioides difficile* infection (CDI) is well-documented [332, 333], its underlying mechanism extends beyond mere bacterial replacement to include the restoration of a complex trans-kingdom ecosystem, involving fungi and viruses that significantly influence therapeutic results.

The gut mycobiome is an emerging determinant of FMT success. Evidence from a large randomized controlled trial indicates that a high pre-FMT abundance of *Candida* is associated with a positive clinical response, while a post-FMT decrease correlates with ameliorated disease severity, suggesting that the baseline fungal state could predict or even modulate the engraftment environment [334]. A potential causal link is supported by experimental data showing that *C. albicans* can reduce FMT efficacy in a mouse model of CDI, and that concomitant antifungal therapy restores therapeutic success [335]. The relevance of trans-kingdom interactions extends to oncology, as highlighted by a meta-analysis demonstrating that fungal and other non-bacterial microbes significantly influence responses to immune checkpoint inhibitor (ICI) therapy, underscoring the broader role of microbial ecosystems in cancer immunotherapy [336].

Arguably, the most influential agents in FMT-mediated ecological restructuring are bacteriophages. The gut virome demonstrates an extraordinary pioneering ability: while transplanted bacterial communities necessitate several months to attain stability, donor-derived phage populations rapidly reestablish themselves in the recipient, often within a span of days [337]. This rapid dominance indicates that phages serve as primary ecological architects in the post-FMT gut. Their proliferation is not a passive occurrence but an active catalyst of microbial dynamics, with the potential to suppress pathobionts and generate ecological niches that support the engraftment of advantageous bacterial taxa from the donor, thereby guiding the entire community toward a state of homeostasis [338–340]. Consequently, the virome should be regarded not merely as a component of the transplant but as a vital initiator and facilitator of microbial restoration.

#### Bacteriophage therapy

Bacteriophages, as natural predators of bacteria, provide an exceptional degree of specificity for the accurate modulation of the gut microbiome, thereby establishing their role as potent therapeutic agents against GI cancers [341]. The advancement of phage-based strategies encompasses various levels of intervention, ranging from ecological restructuring to genetic reprogramming. At the ecological level, phage therapy aims for the targeted eradication of CRC-associated pathobionts such as *F. nucleatum* [342]. While early clinical trials (e.g., NCT03269617, NCT03808103) underscore its translational potential, limitations such as narrow host range and bacterial resistance are being addressed through synthetic biology [343, 344]. Innovative solutions include engineering phage receptor-binding proteins to broaden target specificity [345, 346] and arming phages with CRISPR-Cas systems to confer lethal precision and counteract resistance mechanisms [347, 348]. Beyond bactericidal activity, the field is progressing towards programmable therapeutics. Engineered bacteriophages can be employed as sophisticated delivery vectors to conduct in situ gene editing within the intricate gut microbial community. This methodology leverages tools such as Cas9, dCas9, or base editors to facilitate targeted gene knockout, suppression, or insertion in specific bacterial strains [341, 349]. A significant obstacle in this field remains the limited payload capacity and editing efficiency of phage vectors, which has prompted the development of advanced synthetic biology platforms to surmount these challenges [350]. The therapeutic efficacy of these strategies depends on effective oral administration. Protecting phage viability throughout the GI tract necessitates innovative formulation approaches, such as phage modification [351, 352], advanced encapsulation within pH- or enzyme-responsive materials [353], and sophisticated carrier systems including liposomes or hydrogels to facilitate targeted release within the intestine [354]. The convergence of these approaches demonstrates substantial potential for addressing GI cancers. The paradigm is expanding from simple pathogen clearance to the creation of sophisticated in situ diagnostic and therapeutic systems. For instance, a spheroid-penetrating phage nanovector has been repurposed for the photodynamic therapy of colon cancer, heralding a future where phages serve as versatile platforms for oncology [355]. Realizing this full potential is contingent upon gaining deeper insights into tri-kingdom phage-bacteria-eukaryote interactions and sustaining the current pace of technological innovation.

#### Oncolytic virotherapy

Oncolytic viruses (OVs) are either naturally occurring or genetically engineered viruses that specifically infect and proliferate within malignant cells. These viruses possess the capability to induce oncolysis, trigger immunogenic cell death, and stimulate systemic anticancer immune responses [356]. Oncolytic viruses are a promising immunotherapy class with a unique dual mechanism: direct tumour cell lysis and induction of systemic antitumour immunity. By selectively replicating within cancer cells, OVs trigger immunogenic cell death, remodel the immunosuppressive TME, and act as in situ vaccines, priming T-cells against tumour antigens [357, 358]. This can induce regression of both injected and distant lesions [359]. In the context of GI cancers, clinical data underscore both potential and challenges. In PDAC, a setting renowned for its resistance to immunotherapy, promising indications have arisen. The oncolytic adenovirus CAN-2409, combined with chemoradiotherapy, demonstrated a significant survival benefit (28.8 vs. 12.5 months) in a neoadjuvant phase II trial [360]. Similarly, the reovirus pelareorep combined with chemotherapy and atezolizumab showed a high objective response rate (69%) in a small cohort [361]. However, not all combinations are synergistic. The phase III PHOCUS trial in HCC found that pexastimogene devacirepvec plus sorafenib was inferior to sorafenib alone, underscoring that antagonism with conventional agents is a real risk [362]. Overall, for GI cancers, where immunosuppressive microenvironments are common, OVs offer a strategic tool to “heat up” cold tumours, potentially creating a window of opportunity for enhanced efficacy with checkpoint inhibitors and other immunotherapies [356].

### Modulation of immunotherapy and chemotherapy efficacy

The efficacy of conventional chemotherapy and immunotherapy is profoundly influenced by the gut microbiome. Non-bacterial microbes, including fungi and viruses, have emerged as critical regulators of treatment response, capable of either subverting therapeutic efficacy or serving as powerful allies to enhance it. The gut mycobiome constitutes a significant factor in treatment failure. In cases of PDAC, the elimination of the mycobiome reduces tumour burden and enhances the sensitivity of tumours to gemcitabine chemotherapy [28]. Specifically, *C. tropicalis* mediates chemoresistance in CRC through the downregulation of mismatch repair (MMR) proteins and the upregulation of glycolytic flux [363]. Additionally, this fungal species contributes to CRC progression by augmenting tumour-cell autophagy, a process which results in the downregulation of tumour-intrinsic PD-1, thus providing a mechanistic basis for the limited efficacy of anti-PD-1 therapy in certain patients [364]. Conversely, certain fungi can positively modulate therapy. The presence of *Schizosaccharomyces octosporus* in the GI tract facilitates the fermentation of starch into SCFAs, thereby augmenting the efficacy of ICIs [365], highlighting the potential for harnessing beneficial microbes to boost treatment.

Bacteriophages offer innovative strategies to counteract microbial-driven resistance and improve drug delivery. Phage VA7 effectively surmounts *B. fragilis*-induced resistance to 5-fluorouracil (5-FU) and oxaliplatin in CRC models [257]. Beyond restoring chemosensitivity, bacteriophages are engineered as precise delivery vehicles. A genetically modified, non-pathogenic bacterium can target tumours to release PD-L1-specific M13 phages, and when combined with immunotoxin and FOLFOX chemotherapy, it elicits synergistic antitumour responses [366]. Similarly, the M13@Ag nanoparticle, used with α-PD1 or FOLFIRI, significantly prolongs survival in orthotopic CRC models [259]. The concept of phage-guided drug delivery is further exemplified by Phage A54, a peptide facilitating targeted doxorubicin delivery to HCC, thereby markedly enhancing anti-tumour efficacy [367]. The presence of viruses within tumours can also influence therapeutic outcomes. EBVaGC serves as a paradigmatic example. These malignancies display a remarkable 100% response rate to the anti-PD-1 antibody pembrolizumab, rendering them ideal candidates for immunotherapy, likely attributable to their high PD-L1 expression levels [368, 369]. Although EBVaGCs exhibit resistance to various chemotherapeutic agents [370, 371], such resistance can be mitigated through rational combination therapies, including 5-FU in conjunction with a PI3K inhibitor, or a combination of a PI3K/mTOR dual inhibitor with chloroquine [372, 373].

## Future perspectives and challenges

The emerging field of the non-bacterial microbiome in GI cancers is at a crucial transitional phase, shifting from descriptive ecology to mechanistic and translational research. Over the past decade, it has been conclusively demonstrated that fungi, viruses, and archaea are not merely passive entities but active contributors to oncogenesis. The subsequent challenge is no longer to establish associations but to unequivocally demonstrate causality, analyze the molecular dialogues involved, and utilize this knowledge to transform clinical paradigms in oncology. To achieve this, several significant frontiers and obstacles must be addressed.

Firstly, a technological and analytical renaissance is essential to advance beyond mere correlation. The ongoing controversies, such as the debated fungal signatures in pancreatic cancer, highlight the significant challenge posed by low microbial biomass in tissue samples. Future research must require the adoption of ultra-sensitive, contamination-controlled protocols throughout the entire process, from sample collection to bioinformatic analysis, potentially incorporating synthetic spike-in controls and stringent blank subtraction. Moreover, the field must progress beyond simple taxonomic enumeration. The utilization of multi-omics approaches, including metatranscriptomics to evaluate microbial activity, metabolomics to identify functional effectors, and metaproteomics to analyze the final protein output, will be vital in distinguishing true drivers from incidental passengers. A particularly important direction is the application of spatially resolved approaches to characterize microbial localization within tumours. While bulk sequencing has revealed intratumoural fungal communities in CRC and HCC [65, 92], future studies should employ techniques such as multiplex fluorescence in situ hybridization or laser capture microdissection combined with sequencing to precisely map where fungi (e.g., *Malassezia* species enriched in HCC tumours [92]), viruses (e.g., EBV in GC cells [108–111]), or archaea reside within the TME. Such spatial resolution would distinguish metabolically active microbial “hotspots” from passive contaminants and clarify direct host-microbe interactions at the tumour-immune interface. Integrating these complex, high-dimensional datasets with host parameters (such as immune profiling and host genetics) via advanced machine learning models will be crucial in identifying robust, pan-cancer microbial signatures and in predicting patient-specific microbial contributions to disease. Moreover, the future of this field lies in moving beyond the bulk analyses of homogenized tissue to spatially resolved, high-resolution mapping of microbial communities. Technologies such as spatial transcriptomics and highly multiplexed imaging are poised to revolutionize our understanding [374]. They will enable the direct visualization of fungal hyphae, viral particles, and archaeal biofilms in their native context within the TME. This is crucial for answering fundamental questions: Are specific microbes localized at the invasive front of a tumour? Do they form distinct spatial niches with specific bacteria? How does microbial proximity correlate with localized host gene expression programs? Concurrently, the analysis of bacterial and fungal extracellular vesicles (BEVs/FEVs) represents a groundbreaking new frontier [375, 376]. These nano-sized particles, which carry a diverse cargo of proteins, nucleic acids, and metabolites, can traverse mucosal barriers and enter the systemic circulation. They offer a potential solution to the low-biomass challenge in tissues like the pancreas by serving as circulating biomarkers of the local microbial ecosystem. Furthermore, they represent a novel mechanistic paradigm, as they could act as long-distance messengers, delivering microbial effectors to distant organs and modulating systemic immunity and metabolism. Integrating spatial technologies with the analysis of extracellular vesicles will be essential for establishing causality and translating microbial insights into tangible clinical tools.

Secondly, the fundamental focus of future research must be the unequivocal establishment of causal mechanisms. Although gnotobiotic mouse models have played a crucial role, they frequently fall short of replicating the intricate complexity of human microbial communities. The subsequent generation of research necessitates the development of advanced synthetic ecology models, wherein well-defined, multi-kingdom consortia are introduced into germ-free or humanized mice to analyze the individual contributions of each member and their interactions. Several cross-kingdom interactions described in this review offer testable hypotheses. For instance, the interaction of *Talaromyces islandicus* with *Clostridium saccharobutylicum* and *F. nucleatum* with *Aspergillus rambellii* [64, 242] could be dissected by colonizing germ-free mice with each microbe alone or in combination to assess synergistic effects on tumorigenesis. Similarly, the observation that *C. tropicalis* activates the Syk-PKM2-HIF-1α axis in MDSCs to promote immunosuppression [170–174] can be rigorously tested using defined fungal-bacterial consortia in gnotobiotic settings. The primary yet predominantly unresolved question concerns the nature of the extensive cross-kingdom interactions observed. Are these merely ecological correlations, or do they signify sophisticated, co-evolved syntrophic relationships? Future investigations must strive to delineate the molecular foundations of these networks—whether through metabolic cross-feeding (for instance, bacterial processing of fungal glycans), physical co-aggregation within biofilms, or modulation of shared host immune responses. The ultimate aim is to transition from network observation to a comprehensive understanding of their configuration and to identify key, targetable nodes.

Thirdly, the full translational potential of the non-bacterial microbiome can only be realized through a paradigm shift in therapeutic development. The simple depletion of pro-tumorigenic fungi with antifungals, while informative preclinically, is a blunt instrument fraught with off-target effects and resistance. The future lies in precision targeting. Several examples from the literature illustrate this precision approach. The bacteriophage VA7, which selectively eradicates enterotoxigenic *B. fragilis*, has been shown to restore chemosensitivity to 5-FU and oxaliplatin in CRC models [257]. Similarly, an M13 phage-based nanoprobe targeting *F. nucleatum* has demonstrated efficacy through remodelling of the tumour immune microenvironment in combination with immunotherapy [259]. These examples provide a roadmap for developing other phage therapies against other oncogenic pathobionts, such as phages targeting *C. albicans* or specific CRC-associated *E. coli* strains. The concept of “living therapeutics” will expand to include engineered microbial chassis designed to locally deliver immunomodulatory payloads, degrade carcinogenic metabolites, or even remodel the physical TME. A critical, underexplored avenue is the modulation of the microbiome to enhance existing therapies. Understanding how to strategically manipulate the mycobiome and virome to convert “cold” tumours into “hot” ones, or to reverse chemoresistance, could dramatically improve the efficacy of immuno- and chemotherapy, offering a powerful combinatorial approach.

Finally, the pathway to clinical implementation is characterized by distinctive challenges. The significant inter-individual variability observed in the non-bacterial microbiome necessitates extensive, longitudinal, and meticulously characterized cohort studies to delineate what defines a “healthy” baseline and how it is influenced by factors such as diet, geography, and pharmacological interventions. The regulatory framework for complex biological interventions, including FMT, defined microbial consortia, or engineered phages, remains under development. Prioritizing safety, especially for immunocompromised cancer patients, and establishing standardized manufacturing practices are of utmost importance. Additionally, ethical considerations surrounding the modification of a patient’s intrinsic microbiome demand thorough and careful contemplation.

In conclusion, the future prospects for the non-bacterial microbiome in GI cancers are both challenging and inspiring. By adopting rigorous technological approaches, committing to causal and network-based mechanistic research, and pursuing innovative therapies, it is possible to convert this intriguing ecological observation into a fundamental component of cancer medicine. To achieve this transformation, harnessing advanced computational tools is essential. Artificial intelligence will be instrumental in integrating multi-kingdom omics data, deciphering causal host–microbe interactions, and predicting individualized therapeutic responses, thereby accelerating the translation of microbial insights into clinical practice [377, 378]. The forthcoming era envisions a time when a patient’s distinctive microbial profile not only informs prognosis but also directs a range of targeted, multi-kingdom treatments, thereby ultimately enhancing the precision, efficacy, and personalization of cancer care.

## Data Availability

No datasets were generated or analysed during the current study.
